# Beyond Structure and Change: Ergodicity and the Dynamic Organization of Intellectual Development

**DOI:** 10.3390/jintelligence14100235

**Published:** 2026-10-01

**Authors:** Andreas Demetriou, George Spanoudis, Elena Kazali, Nikolaos Makris, Smaragda Kazi, Alexandra Karousou, Dimitra Economacou, Tryfonas Bikos, Antigoni Mouyi

**Affiliations:** 1 Department of Psychology, University of Cyprus, P.O. Box 20537, 1678 Nicosia, Cyprus; spanoudis.george@ucy.ac.cy (G.S.); antigoni.mouyi@gmail.com (A.M.); 2 Department of Psychology, University of Ioannina, 45110 Ioannina, Greece; ekazali@uoi.gr; 3 Department of Psychology, Democritus University of Thrace, 68300 Didymoteicho, Greece; nmakris@psy.duth.gr; 4 Department of Psychology, Panteion University, 17671 Athens, Greece; kazisma@panteion.gr; 5 Department of Education Sciences in Early Childhood, Democritus University of Thrace, 68100 Alexandroupoli, Greece; akarousou@uniwa.gr; 6 Department of Early Childhood Education and Care, University of West Attica, 12243 Egaleo, Greece; 7 Department of Primary Education, Democritus University of Thrace, 68131 Alexandroupoli, Greece; dimoikonomakou@gmail.com (D.E.); tryfonasibikos@gmail.com (T.B.)

**Keywords:** ergodicity, cognitive development, intelligence, intraindividual change, interindividual differences, developmental dynamics, SARA-C, general intelligence

## Abstract

In developmental research, it is assumed that development is ergodic, i.e., differences between children reveal how individuals change over time. Across four longitudinal studies, we examined ergodicity across cognitive systems—including attention, working memory, reasoning, and cognizance—timescales and developmental phases. We found that development was not globally ergodic. In Study 1, spanning ages 4–17, cross-sectional relations partially resembled patterns of change. Individual development involved overlapping, process-specific periods of acceleration, consolidation, and differentiation rather than one common developmental timetable. Study 2, spanning ages 4–12, showed short-term convergence and partial profile correspondence, with relational integration as the main carrier of change. Study 3, spanning ages 6–13, revealed that psychometric organization was highly stable across three waves, but children changing broadly in the first annual interval were not the same as children changing in the second. In Study 4, 5–7-year-old children were intensively trained on attention control and examined three times on other processes. The trained processes formed a recognizable common organization, but within-person coupling was substantially weaker than between-person coupling, rank correspondence declined with temporal distance between waves, and group dynamics represented individual children only approximately. Across phases, the carriers of change shifted from attention-related control, through relational integration, toward complex reasoning. Psychometric *g*, although strong across time, was very weakly related to developmental g (i.e., phase-sensitive mechanisms transforming states of psychometric g over time), indicating that population-level cognitive organization does not determine individual development. Implications for psychometric and developmental theories are discussed here.

## 1. Introduction

### 1.1. Ergodicity

Developmental science has long relied on a strong assumption: if older children differ from younger children in systematic ways, these differences reflect how children develop. Cross-sectional age gradients, intercorrelations among tasks, and factor structures have therefore been used to characterize performance at a given moment and infer developmental mechanisms. The assumption is that the structure of differences between persons is sufficiently similar to the structure of change within persons to justify moving from one to the other. In formal terms, it is assumed that development is ergodic (Curran & Hancock, 2021; Molenaar, 2008, 2010).

The issue is not merely technical. If development was ergodic, cross-sectional and longitudinal observation would become interchangeable, and group-level patterns of performance would stand for developmental processes; if development was non-ergodic, that shortcut breaks down. Between-person structures may then describe who differs from whom, but not how development unfolds within the individual. A middle-ground position assumes that an average group-based developmental trajectory may describe a population well without necessarily describing any child well. In this case, person-specific information may be needed to account for the state and change of specific individuals (Hunter et al., 2024; Voelkle et al., 2014). This is especially important in cognitive development, where maturation, learning, strategy changes, reorganization, and reflection all produce dynamics that are unlikely to be stationary, homogeneous, or linear.

Strict ergodicity refers to equivalence between time- and ensemble-level statistics under stationarity and homogeneity. The present study does not assume that these demanding conditions hold globally; rather, it examines several distinct empirical implications of cross-level equivalence—dispersion balance, rank persistence, structural profile correspondence, structural magnitude discrepancy, developmental change correspondence, and time-average convergence. These indices answer different questions and are not treated as interchangeable estimates of one latent construct. The studies presented here provide empirical evidence directly related to all of these forms of ergodicity.

This paper synthesizes four developmental studies differing in age range, measurement density, and cognitive content. Studies 1 and 2 included two observed annual waves over broad age spans and theory-guided ten-wave extensions. Study 3 included children observed annually on three occasions in middle childhood, allowing direct comparison of two real developmental intervals and prediction of a real third wave. Study 4 included 30 sessions of attention training from 5 to 7 years and a broader cognitive battery administered at Sessions 1, 15, and 30. Together, the studies permit comparison of cross-sectional organization, longitudinal change, person-level correspondence, and the consequences of measurement density.

Altogether, these studies provide a broad basis for examining where cross-level correspondence is stronger or weaker and what this implies for theories of intelligence and development. This study has the four following aims: First, it assesses distinct implications of ergodicity across four developmental studies within a common comparative framework. Second, it examines how correspondence varies across cognitive systems and individuals, including whether person-level correspondence is temporally stable and predicts subsequent development or learning. Third, it evaluates the conditional value and limitations of extended developmental modeling. Fourth, it examines how the number and spacing of observations affect conclusions about developmental coupling, transition timing, and group-to-individual generalizability.

### 1.2. Theory-Based Predictions

The history of developmental psychology involves a fundamental tension between the search for universal developmental patterns and the recognition of individual variability. The concept of ergodicity bridges these aspects of development because it can express how much population-level developmental organization generalizes to individual trajectories. Theories differ in their assumptions; for example, Piaget’s (1970) theory emphasizes sequentially invariant, hierarchically integrated, and universal stages, implying that age-related differences broadly reflect within-person developmental succession, while Vygotsky’s (1978) theory allows for greater variability because cultural and social influences shape when and how individuals realize their mental possibilities, with the zone of proximal development reflecting the dynamic and relational nature of cognitive change.

More recent theories are more flexible. Information processing theories conceptualize development as gradual improvement in cognitive efficiency through domain-general mechanisms, such as learning and representational capacity, implemented through domain-specific learning that may produce variability between individuals (Case, 1992; Ferrer & McArdle, 2010; Kail, 1991; Siegler, 1996). Theories invoking core processes (Kovacs & Conway, 2016) or interactions among processes (van der Maas et al., 2017) also allow for process- and environment-dependent variation in cross-level correspondence.

**Figure 1:**
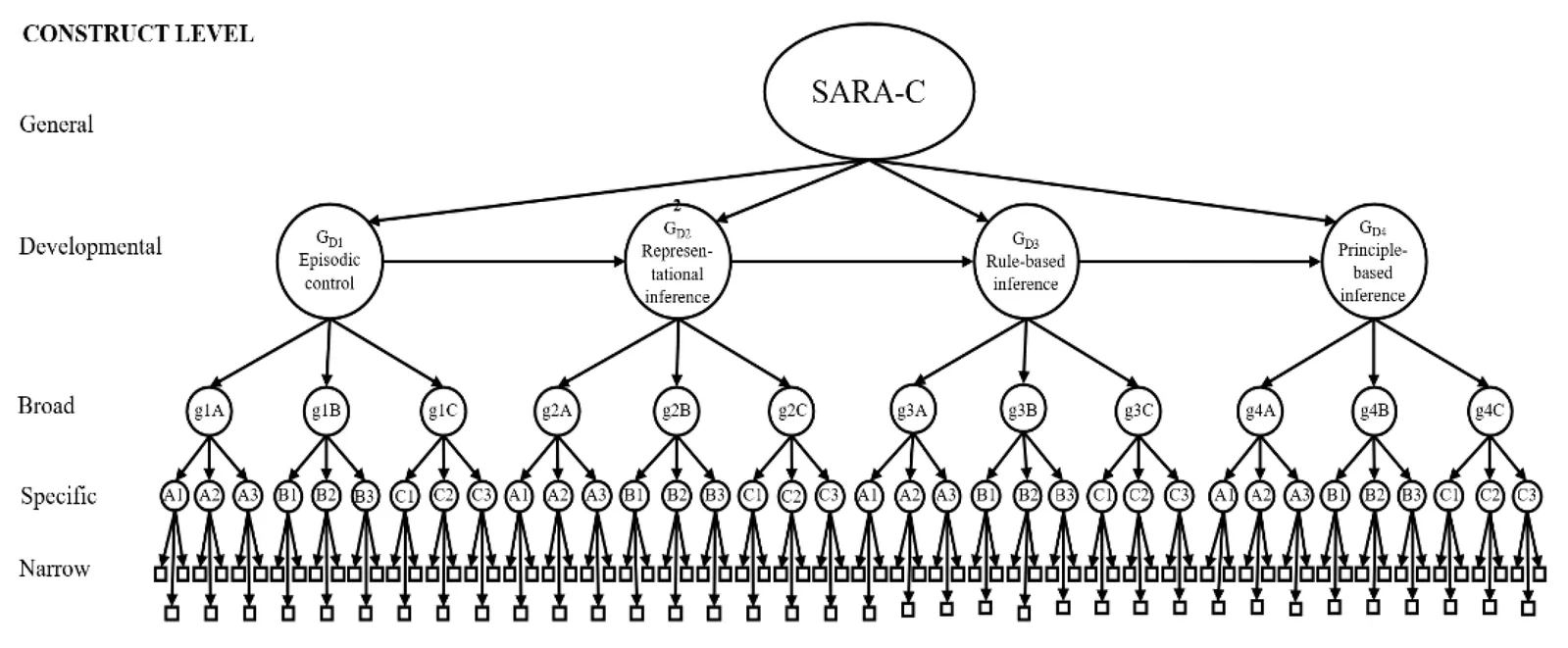
The hierarchical structure of mind, including core SARA-C processes, developmentally specific g factors, and domain-specific reasoning systems. The figure presents the theoretical architecture motivating the analyses; the present results do not uniquely identify this architecture.

This study explores ergodicity from the perspective of Developmental Priority Theory (DPT; Demetriou et al., 2026), which integrates developmental, psychometric, and cognitive research and assumes that different cognitive processes become functionally dominant during different developmental phases. The basic postulates are as follows:

1. In evolution and development, intelligence varies along a dimension of complexity control enabling organisms to integrate information and action across increasingly diversified perceptual and action systems, thus enhancing their adaptive efficiency (Coombs & Trestman, 2025; Demetriou et al., 2025; Woodley of Menie & Peñaherrera-Aguirre, 2022).
2. This dimension draws on a core meaning-making mechanism, SARA-C, integrating the perceptual, inferential, and awareness processes Search, Align, Relate, Abstract, and Cognize. SARA-C is proposed as a recursive inferential mechanism underlying psychometric g, enabling goal-related exploration, relational integration, monitoring, and self-regulation across evolution, development, and artificial intelligence (Demetriou et al., 2025).
3. SARA-C operates as a Bayesian generative system that iteratively updates models of the world as new evidence arrives, building increasingly abstract, self-aware, and self-regulated systems of understanding. Cognitive processes thereby become deeper when uncovering relations, broader when generalizing learning outcomes, and more flexible when managing variation (Demetriou et al., 2025, 2026).
4. Eight levels are described, ranging from reflexive control and associative learning to hierarchical predictive control. In humans, development follows four major levels, from action control in infancy to representational control in early childhood, inferential control in middle and late childhood, and truth or principle control in adolescence. Attention control and representational awareness dominate early childhood; relational integration, inductive reasoning, and inferential awareness become central in middle childhood; advanced deductive and domain-specific reasoning and awareness of cognitive processes become increasingly important in adolescence (Demetriou et al., 2024, 2025, 2026).
5. SARA-C is proposed to underlie evolutionary and psychometric general intelligence. Evidence from previous work indicates that developmentally changing SARA-C processes shape how psychometric g is expressed over domains and ages (Demetriou et al., 2024, 2025). This hierarchy is shown in Figure 1.
6. Assuming SARA-C does not imply uniform performance across species, individuals, and domains. Genetic, neural, environmental, educational, and cultural factors influence how SARA-C possibilities translate into learning, reasoning, and problem-solving. Some domains, such as inductive and deductive reasoning, may express central mechanisms more directly than others, such as mathematics (Demetriou et al., 2026).

Rather than implying uniform developmental trajectories, this perspective highlights dynamic reorganization over time. DPT therefore suggests that ergodicity is unlikely to be uniform across age or domain, and should be treated as a graded and developmentally contingent property rather than a binary characteristic. Two predictions follow:

1. Early developmental phases are characterized by high variability and rapid reorganization, weakening the correspondence between within-person and between-person structures. Periods of consolidation may temporarily increase alignment; later differentiation may again reduce correspondence as strategies, choices, and experience diversify.
2. Developmentally central processes, such as attention control and relational integration, may show stronger cross-level correspondence during periods of developmental dominance than during emergence or later differentiation. Other processes, including cognizance and complex domain-specific knowledge, may reorganize in relation to the processes currently assuming priority.

Non-ergodicity does not imply the absence of lawful structure; rather, it indicates that the laws of intraindividual change are not directly recoverable from interindividual variation at one time. Developmental organization may be systematic while unfolding along individual-specific trajectories shaped by prior states, constraints, regulatory mechanisms, and experience. For instance, temporal desynchronization between individuals is one possible source of non-ergodicity, reflecting different developmental paces along the same course. Developmental degeneracy—that is, the mind’s ability to reach a developmental end-state by alternative paths—is another cause of non-ergodicity (Di Plinio et al., 2024). Ergodicity is therefore treated here as a diagnostic indicator of developmental constraint, where higher correspondence indicates stronger convergence around a shared organization while lower correspondence indicates emergence, transition, differentiation, or flexible realization within that organization. For the first time, the present study presents extensive longitudinal empirical evidence directly related to the various forms of ergodicity in cognitive development from early childhood to late adolescence.

At the individual level, DPT implies that conformity to the age-normative organization may be stronger when a child is moving through a relatively canalized phase and weaker during reorganization or differentiation. Such correspondence need not be a fixed personal characteristic, nor does it necessarily imply higher ability or faster development. Therefore, a critical test is whether individuals who resemble the group organization at one interval retain that relative position, and whether early correspondence predicts subsequent change.

In summary, this study advances the following central claim: cognitive development is governed by common constraints whose expression is selectively ergodic and increasingly differentiated across development. Understanding this pattern requires moving beyond simple equivalence between structure and development toward explicit comparison of the two.

## 2. Materials and Methods

The four studies presented below are ordered by age coverage and measurement design. Study 1 spans approximately 4–17 years at the first test and assesses attention, working memory, multiple reasoning domains, cognizance, and awareness of cognitive processes. Study 2 spans approximately 4–11 years and adds relational integration tasks. Study 3 is a three-wave annual study of children beginning at approximately 6–11 years, included to test whether developmental change organization reproduces across two real intervals and whether models fitted to the first transition predict the observed third wave. Study 4 combines a 30-session attention training program spanning from 5 to 7 years, with broader cognitive assessments at Sessions 1, 15, and 30. The observed and model-generated parts of each dataset are reported separately.

Ergodicity was assessed at four complementary levels: variability, structural, dynamic, and averaging ergodicity (Molenaar, 2010). These labels refer to distinct empirical implications of cross-level correspondence, rather than interchangeable measures of strict ergodicity. Table 1 provides descriptive interpretive ranges, which are heuristics only; continuous estimates, uncertainty intervals, magnitude discrepancies, and matched-null comparisons carry the primary interpretation. Each form of ergodicity is formally defined in Supplementary S1.

*Variability Ergodicity:* This concerns the relation between variability within individuals across time and variability between individuals in a developmental period. The theoretical parity target is equality of time and ensemble variance (Molenaar et al., 2003). In Studies 1 and 2, rank stability, an ICC-like stable component, and a within/between variance ratio are reported separately because they answer different questions. Study 3 distinguishes stability across the two adjacent annual intervals from stability across the complete two-year lag. In Study 4, the intensive series calculation uses within/between standard deviation ratios; these are explicitly distinguished from variance ratios.

*Structural Ergodicity:* This concerns correspondence between the organization of cognitive processes across individuals and their organization within individuals across 30 repeated observations (Voelkle et al., 2014). The relative profile is summarized by the correlation between unique off-diagonal matrix elements; absolute discrepancy is summarized using the Mean Absolute Discrepancy (MAD). In Studies 1 and 2, two-wave matrices based on change scores are described as cross-level developmental change correspondence rather than person-specific temporal covariance. In Study 3, a pooled three-wave within-person matrix was estimated from repeated deviations around each child’s own mean; as such, it remains a pooled repeated-measures quantity rather than a person-specific time-series model.

*Dynamic Ergodicity:* This concerns the correspondence between cross-sectional cognitive organization and the organization of developmental increments. High profile correspondence must be interpreted together with mean coupling and MAD; similar ordering does not imply equal magnitude or a common causal mechanism (Voelkle et al., 2014).

*Averaging Ergodicity:* This asks whether cumulative individual histories become increasingly similar to the ensemble trajectory. As finite autocorrelated series do not converge to zero exactly, observed dispersion is compared with a matched finite-sample autoregressive null where possible (McLeish, 2015).

Sampling uncertainty was evaluated by resampling complete participants, preserving all observations belonging to each selected child. At least 1000 bootstrap replications were used for primary observed-data diagnostics and 2000 for gain-window and several contrast analyses. Whole-matrix uncertainty was assessed by recalculating complete matrices under subject-level resampling and exact or Quadratic Assignment Procedure (QAP)-style joint row-and-column permutations, avoiding the assumption that off-diagonal cells are independent. Benjamini–Hochberg false discovery rate correction was applied within prespecified families of related tests; both unadjusted *p* values and adjusted q values are supplied in the Supplementary Materials.

Score-level reliability, empirical floor/ceiling concentration, and cross-wave correlation structure comparability were examined where the available composite files permitted; these checks do not substitute for item-level longitudinal measurement-invariance analysis. Predictive validation used held-out observed outcomes; that is, nested fivefold prediction of Wave 2 in Studies 1 and 2, prediction of the real Wave 3 from models estimated on Waves 1–2 in Study 3, and prospective prediction of Sessions 16–30 in Study 4. Synthetic participants were never treated as additional independent empirical observations. Conditional intervals for the selected model panels used donor/source-lineage resampling and are explicitly distinguished from sampling confidence intervals and from uncertainty across model refits, seeds, and hyperparameters. Full details of participant-level resampling, whole-matrix permutation, false discovery rate correction, spline specification, measurement diagnostics, and predictive validation are provided in Supplementary S3.

Person-level correspondence was examined with design-specific indices. In the conditional Study 1 panel, a leave-one-out Wave-1 correlation profile among the eight cognitive families defined the reference structure for each starting-age cohort. For participant i, the correlation of that reference with the individual ten-wave family-pair profile (rSi) and with the individual nine-increment profile (rΔi) was Fisher-z-averaged and mapped to a 0–100 score as follows: Ii = 50{1 + tanh[(z(rSi) + z(rΔi))/2]}. Continuous scores were used for inference; bottom, middle, and top quintiles were used only to display profile differences. In Study 3, each child’s nine-task change vector was compared via cosine similarity with the leave-one-out cohort mean change vector in each annual interval. In Study 4, P-technique loading congruence during Sessions 1–15 was compared with congruence during Sessions 16–30 and tested prospectively as a predictor of the later composite learning slope, controlling early slope, Session-15 level, age, and gender. These indices describe individual cross-level correspondence; they are not trait measures of strict ergodicity.

The studies were conducted in Greek-speaking educational settings, which are generally representative of Western populations; hence, general claims concern the developmental patterns observed in these samples and require replication across languages, educational systems, and cultures.

The extended datasets were not treated as simple extrapolations; their purpose was to recover latent developmental ordering from sparse longitudinal data while preserving observed tendencies. Supplementary S2 explains the extension implemented in Study 1; extension in the other studies was based on the same principles. Recursive extension can clarify conditional developmental consequences, but it may also overestimate coherence, smooth individual differences, and carry the initial observed derivative forward. Provenance, age-support, score-support, and post-generation sensitivity checks were therefore included. Full operational definitions and study-specific results for these person-level correspondence indices are reported in Supplementary S8 (Tables S26–S29; Figures S1 and S2).

## 3. Results

### 3.1. Study 1: A Comprehensive Study of Cognitive Processes from Early Childhood to Late Adolescence

Study 1 was the broadest study in the project; it examined 425 participants aged approximately 4–17 years twice, at an interval of about one year. The participants were recruited from public schools in Alexandroupolis and Athens, Greece, and were tested between December 2021 and May 2022. Parental education and socioeconomic status (SES) were broadly representative of the Greek urban population, with ~50% of both parents having a university degree. Participants were tested on attention control (shifting and inhibition), working memory (serial recall of words and pseudowords and recall of spatial positions), deductive reasoning (modus ponens, modus tollens, affirming the consequent and denying the antecedent), Raven-based inductive reasoning, problem-solving in mathematical and spatial tasks, and cognizance (perceptual and inferential cognizance, and awareness of cognitive processes). Participants and task batteries are described in the work of Demetriou et al. (2026). The observed analyses used the 425 original participants and the two real waves. Reliability at Wave 1 was generally high across task families, whereas reliability of change varied substantially. Detailed reliability, score boundary, resampling, matrix permutation, and held-out-prediction results from Study 1 are reported in Supplementary S4 (Tables S2–S4).

**Figure 2:**
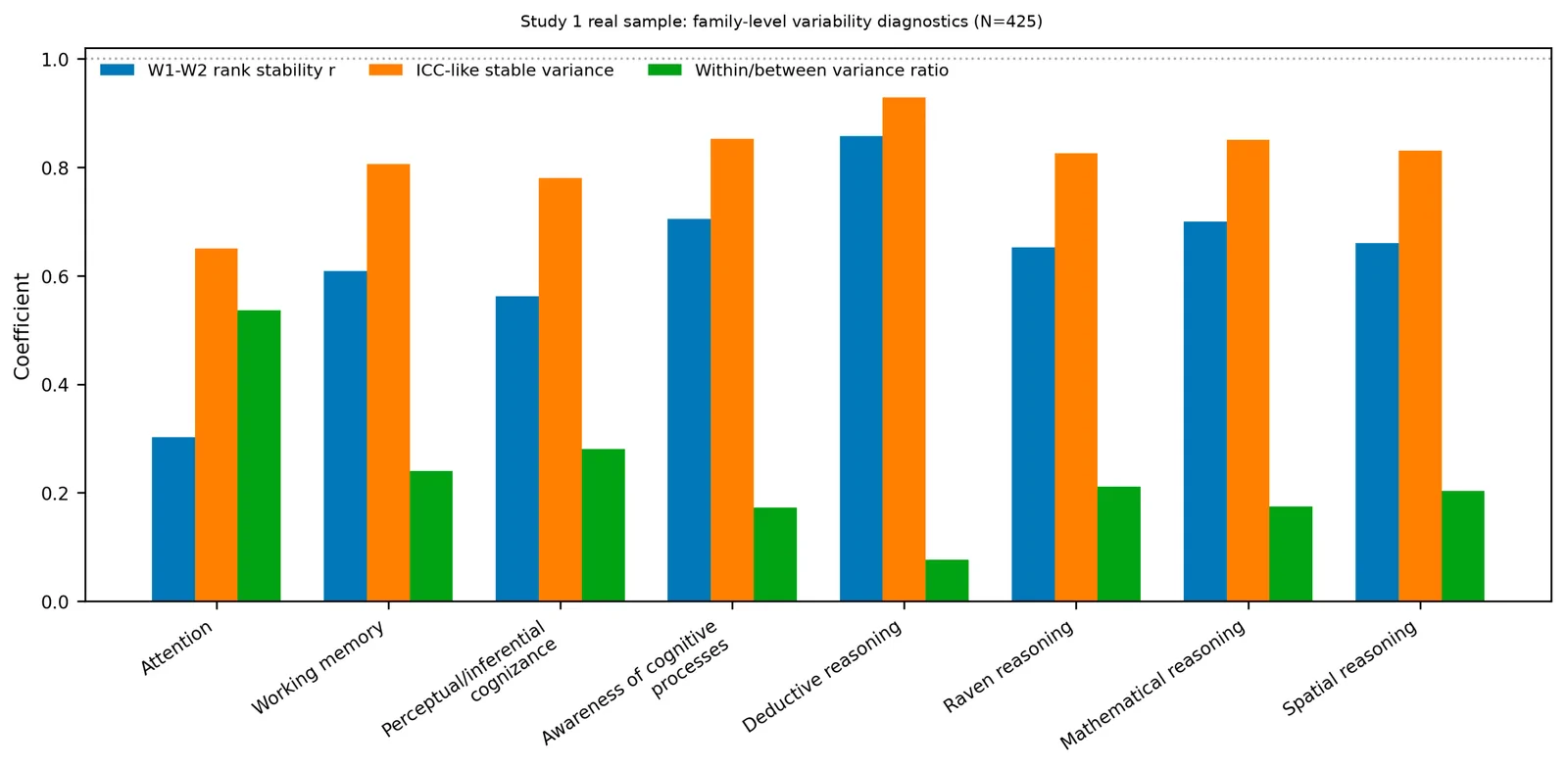
Family-level variability diagnostics in the real Study 1 sample. Bars show Wave 1–Wave 2 rank stability, the ICC-like stable-variance coefficient, and the within-person–between-person variance ratio. These are distinct diagnostics and are interpreted continuously.

*Variability Ergodicity:* In the real sample, the Wave 1–Wave 2 rank stability was 0.303 for attention, 0.609 for working memory, 0.563 for perceptual/inferential cognizance, 0.705 for awareness of cognitive processes, 0.858 for deductive reasoning, 0.653 for Raven reasoning, 0.700 for mathematical reasoning, and 0.661 for spatial reasoning (see Figure 2). Age adjustment altered the interpretation markedly; deductive reasoning fell to 0.210 and attention to 0.047. Much of the impressive raw stability over this wide age span therefore reflected normative age ordering rather than equally strong rank persistence among children at comparable ages. Working memory, cognizance, awareness of cognitive processes, mathematics, and spatial reasoning retained moderate age-independent stability.

Participant-level bootstrap analyses confirmed that the raw-to-adjusted reduction was reliable in all eight families (all q = 0.001). Adjusted attention stability was not distinguishable from zero (95% CI [−0.040, 0.128], q = 0.338), whereas the remaining adjusted coefficients were positive. Within/between variance ratios ranged from 0.077 for deductive reasoning to 0.537 for attention, and were below parity in every family (all q = 0.001). These results support local but strongly age-graded continuity, rather than time-invariant individual ranking.

**Figure 3:**
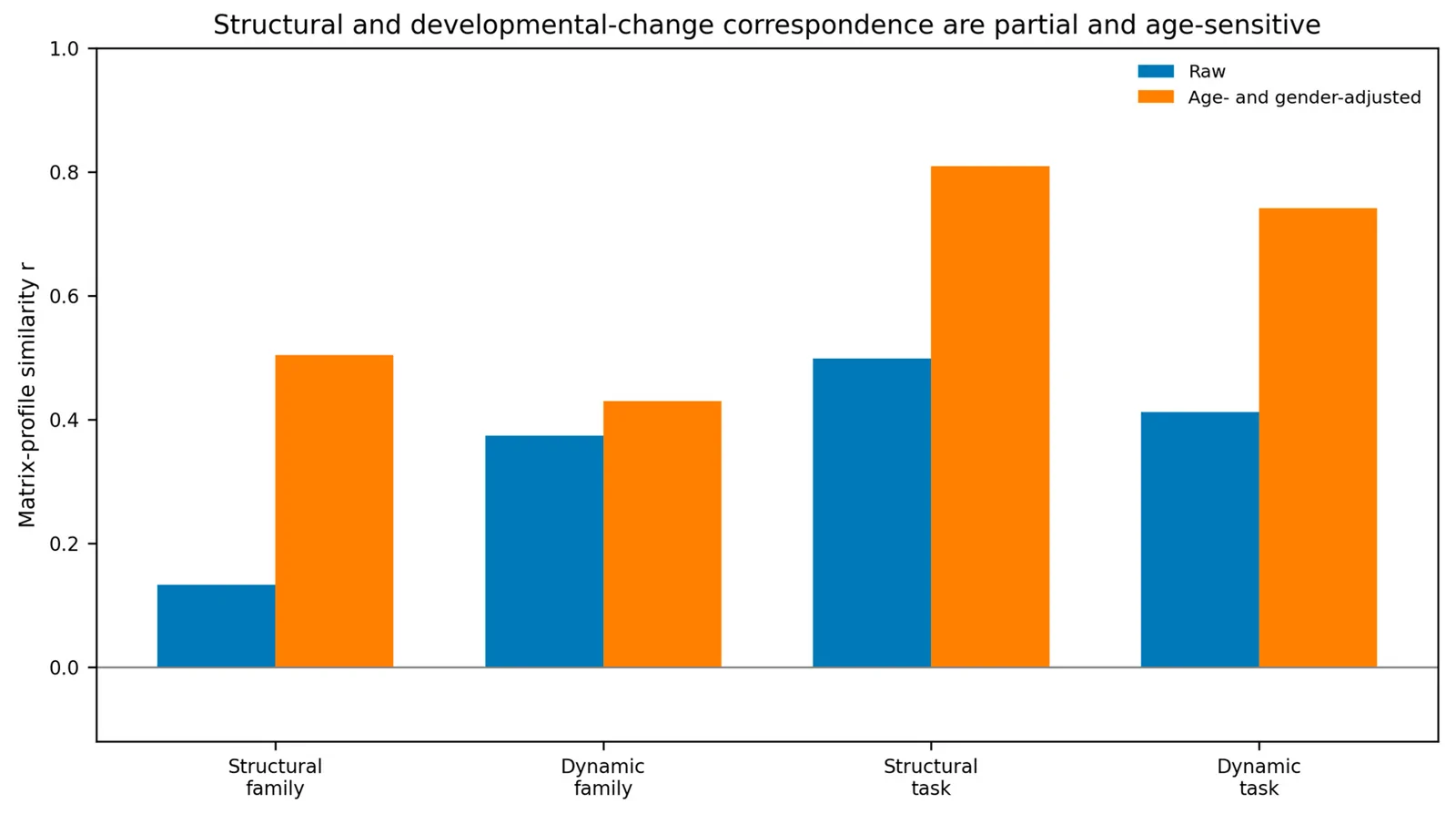
Structural and developmental change profile similarity before and after removal of age and gender. Adjustment increases profile correspondence, especially at the task level, but does not imply equality in coupling magnitude.

*Structural Ergodicity:* The raw family-level between-person structure was highly integrated—the mean cross-sectional family correlation was 0.541, whereas the mean one-year change coupling was 0.147. Matrix profile similarity was rS = 0.133 and MAD = 0.395. After age and gender were removed, family-level profile similarity increased to rS = 0.504 and MAD fell to 0.102. At the task level, profile similarity increased from 0.498 in the raw analysis to 0.809 after adjustment, with MAD falling from 0.298 to 0.068. The mean adjusted between-person and developmental change task correlations remained weak, reaching approximately 0.155 versus 0.055 at the family level and 0.099 versus 0.036 in the original task-level summary (see Figure 3).

Whole-matrix inference supported the adjusted profile findings. Adjusted family-level structural correspondence had q = 0.013; adjusted task-level correspondence had r = 0.809, 95% bootstrap CI [0.767, 0.852], QAP q < 0.001. Thus, the relative ordering of task relations was robustly similar after age adjustment, while the magnitude of developmental coupling remained much weaker than population-level coupling.

*Dynamic Ergodicity:* Dynamic correspondence was assessed by comparing the Wave-1 between-person matrix with the matrix of W1–W2 developmental changes across children. At the family level, the raw profile similarity was rD = 0.374 with MAD = 0.534; the mean Wave-1 family correlation was 0.608, whereas the mean correlation among one-year changes was 0.073. Age adjustment increased task-level profile similarity from 0.412 to 0.741 and reduced MAD from 0.369 to 0.071. These are cross-level change profile results, not person-specific time-series covariance estimates (see Figure 3).

The adjusted family-level dynamic profile was reliable under exact whole-matrix permutation (r = 0.429, q = 0.029); the adjusted task-level result was r = 0.741, 95% CI [0.684, 0.798], QAP q < 0.001. These tests support the profile-versus-magnitude conclusion while making its uncertainty explicit.

**Figure 4:**
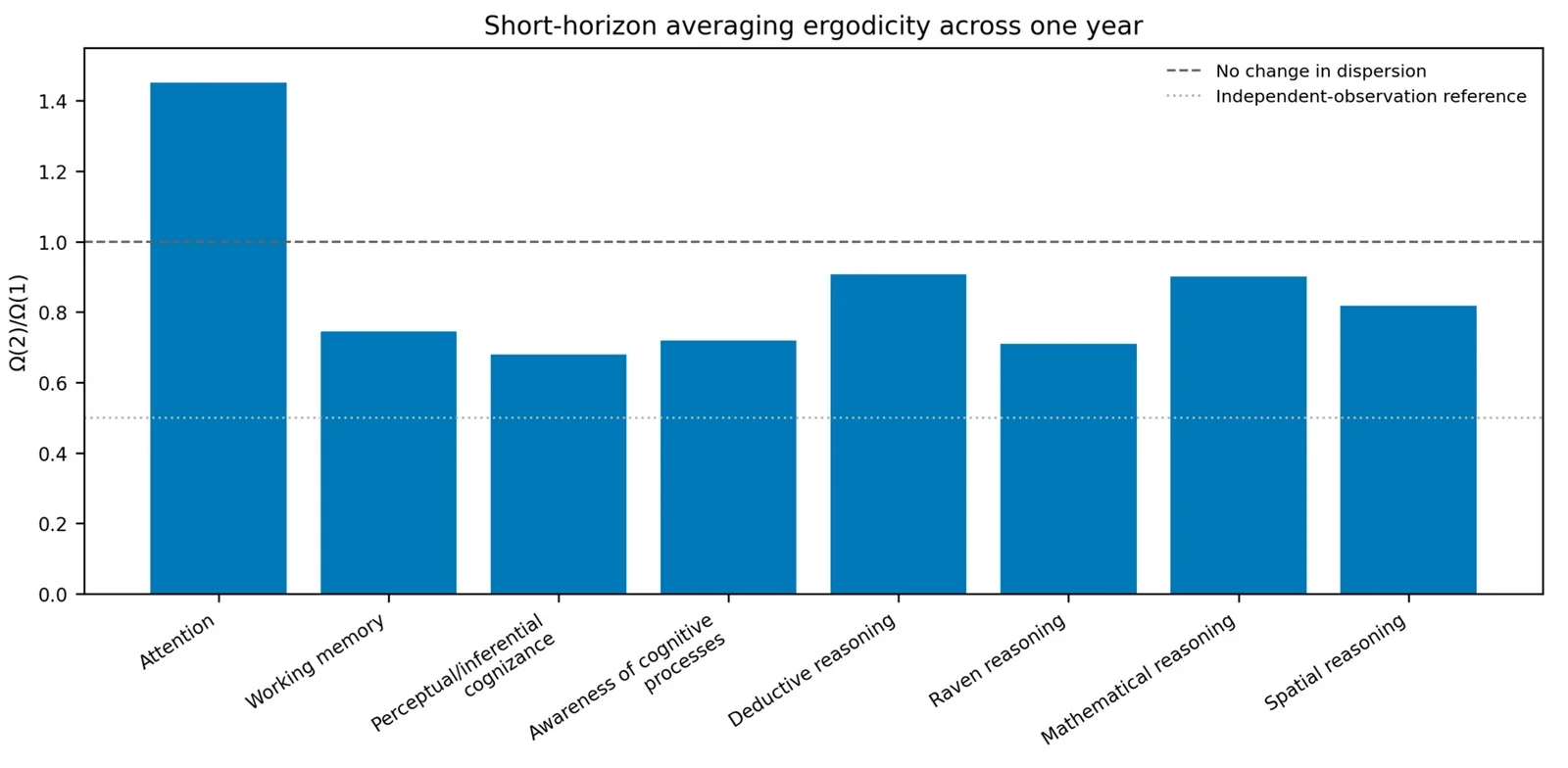
Short-horizon averaging ergodicity across one year. Values below 1 indicate convergence, and values above 1 indicate differentiation; attention is the clear exception.

*Averaging Ergodicity:* Most families showed a reduction in cumulative dispersion over the one-year interval. The strongest convergence occurred in perceptual/inferential cognizance (RΩ = 0.679), Raven reasoning (0.710), awareness of cognitive processes (0.720), and working memory (0.744). Spatial reasoning showed weaker convergence (0.819), and deductive and mathematical reasoning were close to stable dispersion (0.907 and 0.900, respectively) (see Figure 4).

Attention’s cumulative dispersion increased from Ω(1) = 0.382 to Ω(2) = 0.555, giving RΩ = 1.452. The bootstrap interval [1.247, 1.695] excluded parity, and the FDR-adjusted test was significant (q = 0.001). Thus, attention differentiation is unlikely to be due to sampling fluctuation within the observed interval. The two-wave design nevertheless cannot show whether it persists or represents one phase of a longer cycle.

Measurement checks qualified some change results; in particular, change score reliability was high for attention but very weak for working memory and spatial reasoning, and several bounded tasks showed floor or ceiling concentration. These properties may attenuate developmental change correlations. Nested cross-validation predicted held-out Wave 2 with family correlations from 0.357 to 0.924 and standardized RMSE from 0.934 to 0.381. This supports one-year prediction from the observed data but does not validate recursive Waves 3–10.

#### 3.1.1. The Extended Sample

The extended sample retained the 425 original children and added 875 synthetic cohort-balancing cases, yielding 100 cases at each starting age from 4 through 16 years, with a model-based N = 1300. Observed Waves 1 and 2 were retained for the original participants; Waves 3–10 were generated. All ten waves were generated for fully synthetic participants; the panel is therefore a model-based developmental laboratory, not a ten-wave observed longitudinal study. The findings describe the consequences of the fitted age functions, one-year transitions, phase shifts, innovation structure, and donor interpolation.

**Figure 5:**
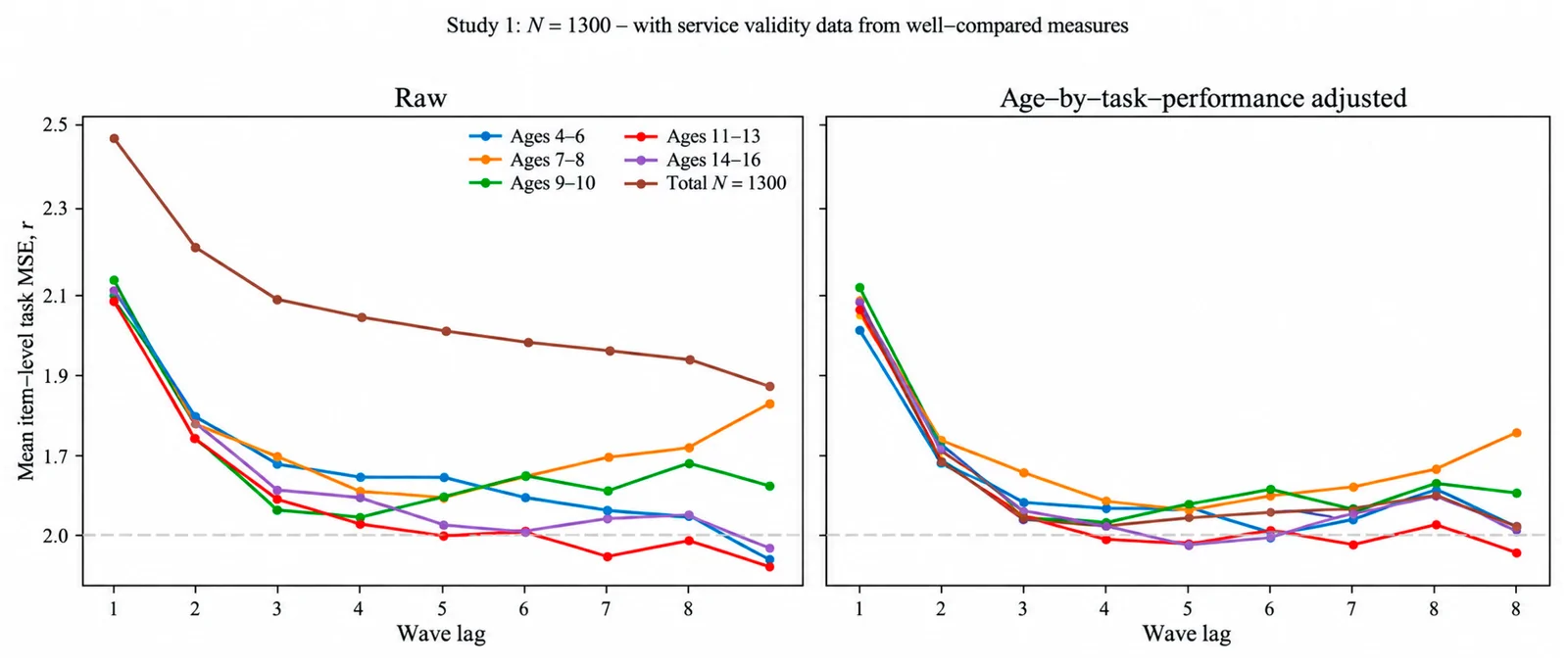
Mean family rank-order stability as a function of temporal lag. The left panel shows raw scores; the right panel removes nonlinear current-age trends, gender, and real-versus-synthetic provenance. All later-wave results are conditional on the selected generative panel.

*Variability Ergodicity:* In the total panel, the mean family rank stability declined from 0.483 at adjacent waves to 0.183 across nine waves. Adjustment for current age, gender, wave, and provenance reduced adjacent stability to 0.268 and lag-9 stability to 0.009, with a source-lineage interval of [−0.017, 0.035]. Within the five narrower developmental periods, adjusted lag-9 stability was near zero or negative except in ages 7–8; broad-age stability therefore did not imply preservation of age-local ordering (see Figure 5).

**Figure 6:**
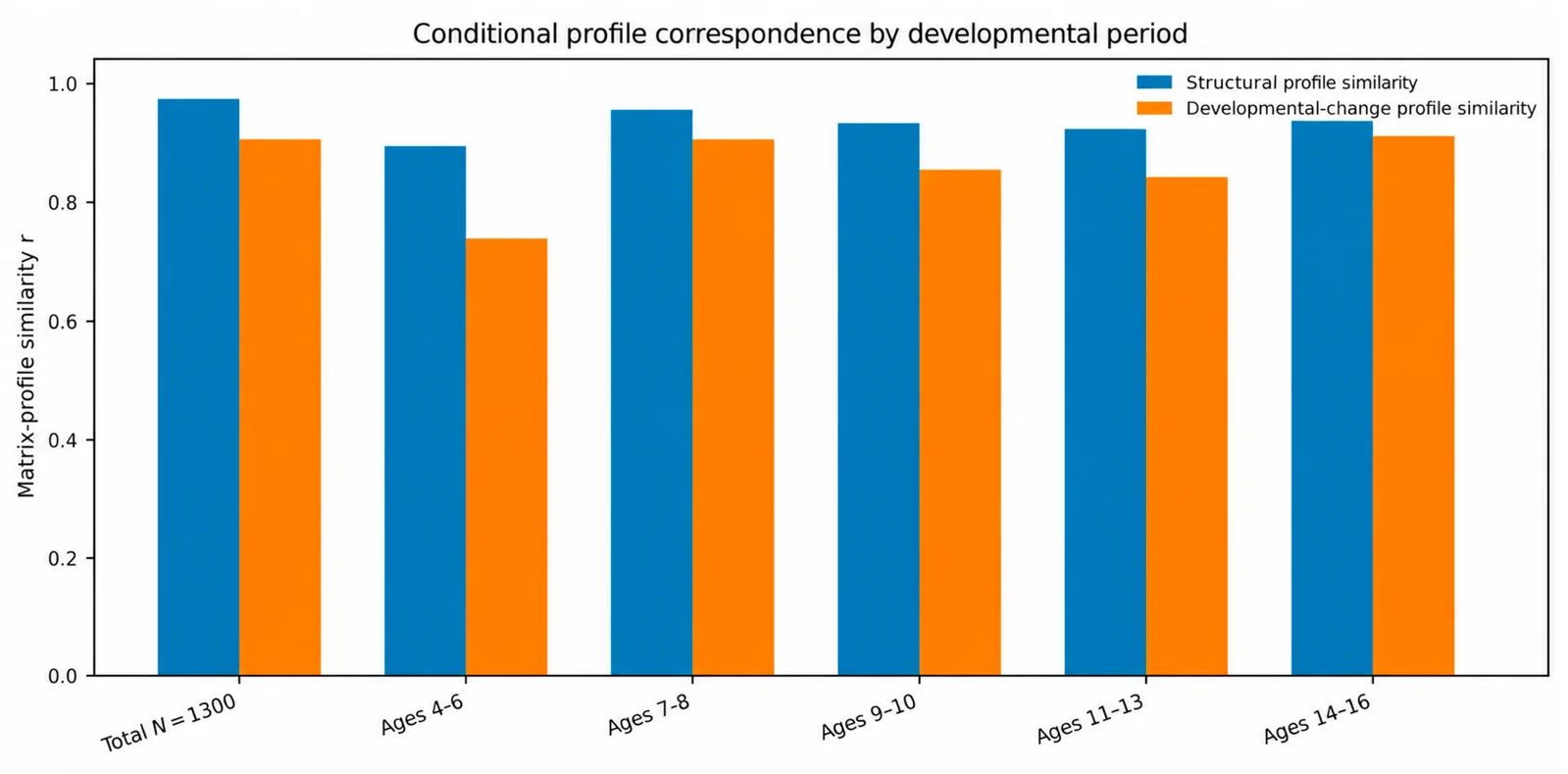
Conditional structural and developmental change profile similarity after adjustment for nonlinear current age, gender, and data provenance. These model panel coefficients are not independent empirical replications.

*Structural and Dynamic Ergodicity:* After adjustment, family-level structural profile similarity was 0.972 in the total panel and 0.893–0.954 across developmental periods; task-level similarity was 0.987 overall. Family-level developmental change profile similarity was 0.904 overall and 0.736–0.908 across periods; task-level similarity was 0.968 overall. The mean adjusted correlations remained small; thus, the selected panel shows high relative profile preservation but weak magnitude equivalence, and the profile correspondence is partially inherited from an architecture designed to preserve empirical covariance and change patterns (see Figure 6).

**Figure 7:**
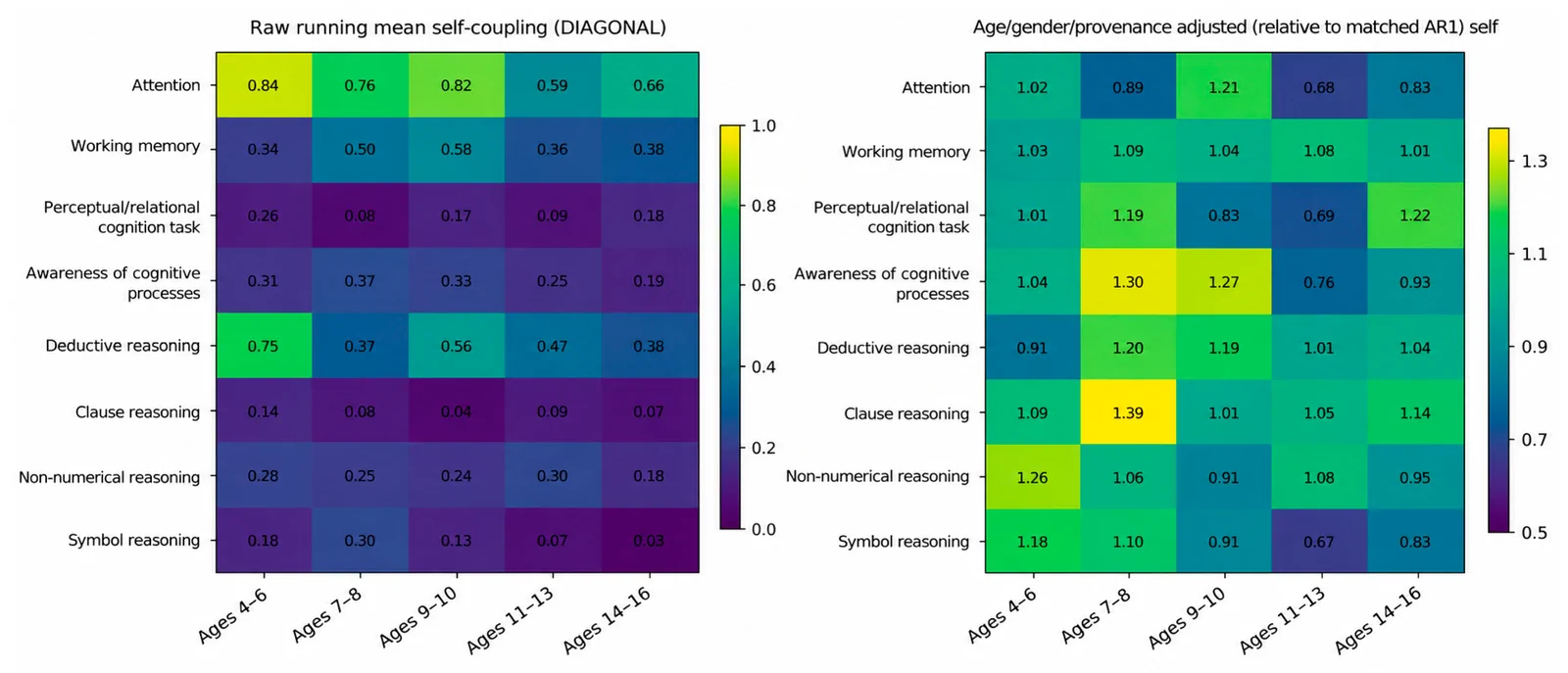
Averaging ergodicity in Study 1: The left heatmap shows raw running-mean convergence; the right compares age-adjusted terminal dispersion with a matched homogeneous AR(1) expectation. Values are conditional on the selected panel.

*Averaging Ergodicity:* Every family had a raw terminal ratio below 1; the mean Ω(10)/Ω(1) ratio was 0.218 in the total panel. After detrending and normalization, the mean terminal observed-to-matched-AR(1) ratio was 1.029, with a source-lineage interval [0.998, 1.058]. There was therefore no universal excess over the matched autocorrelated expectation, although selected family–period combinations differed from parity (see Figure 7).

Provenance analyses showed that fully synthetic cases had slightly higher raw adjacent-wave stability than the original-participant projected trajectories (difference = 0.050), consistent with smoother interpolation; however, they had slightly lower adjusted structural and developmental change profile correspondence. These comparisons indicate that the synthetic cases did not create stronger adjusted correspondence, but did alter the local smoothness. Full generator sensitivity to model class, seeds, blending weights, and perturbation parameters still requires the original generation ensemble.

#### 3.1.2. Critical Windows of Change: Process-Specific Developmental Windows

The two-wave design precluded the identification of a sequence of person-specific transition windows inside each child, but did enable estimation of the starting age at which each process shows its largest baseline-adjusted one-year gain. For each task, Wave-2 performance was modeled as a function of Wave-1 performance, a cubic B-spline of starting age, and gender as follows:

*X_i_*_2_
= *α* + *βX_i_*_1_ + *s*(*Age_i_*_1_
) + *γGender_i_* + *ε_i_*

The primary spline used five degrees of freedom. The expected one-year gain at age a, peak age, and 80% high-gain window were defined as follows:

$$G_{p}(a)\;=\;{\hat{X}}_{p,2}(a)\;-\;{\hat{X}}_{p,1}(a),\;{a^{*}}_{p}\;=\;\underset{a}{arg\;max}\;G_{p}(a),\;W_{p}\;=\;\left\{a:G_{p}(a)\;\geq \;0.80G_{p}\left({a^{*}}_{p}\right)\right\}$$

All eight family-level age functions remained significant after FDR correction. Peaks at the youngest or oldest observed age were boundary estimates. Sensitivity analyses repeated the spline with 3–7 degrees of freedom and 2000 participant bootstraps. The attention peak near age 6.7 was comparatively robust; several other family peaks were boundary-dominated, broad, or multimodal. These results are therefore interpreted as age-specific maxima in one-year gain, not observed within-child tipping points (see Table 2).

The family-level results identify a broad developmental sequence, but the windows are not mutually exclusive stages. Several systems reorganize in parallel, and growth continues outside each high-gain interval. The timing of maximum relative one-year gain differs across processes; only the attention window was compact and comparatively robust to spline specification.

#### 3.1.3. Individual Differences in Ergodicity and Their Implications for Development

System-level coefficients may conceal substantial differences in how closely individual developmental trajectories reproduce the organization observed in their age group. Because only two waves were observed in Study 1, this person-level analysis used the selected conditional ten-wave panel and must be interpreted as a model-based test. For each starting-age cohort, a leave-one-out Wave-1 correlation profile among the eight cognitive families served as the cross-sectional reference. Each participant’s ten-wave family-pair profile and nine-increment profile were correlated with that reference, Fisher-z-averaged, and converted to a 0–100 individual cross-level correspondence score. The structural and increment components were related (r = 0.694) but not redundant.

**Figure 8:**
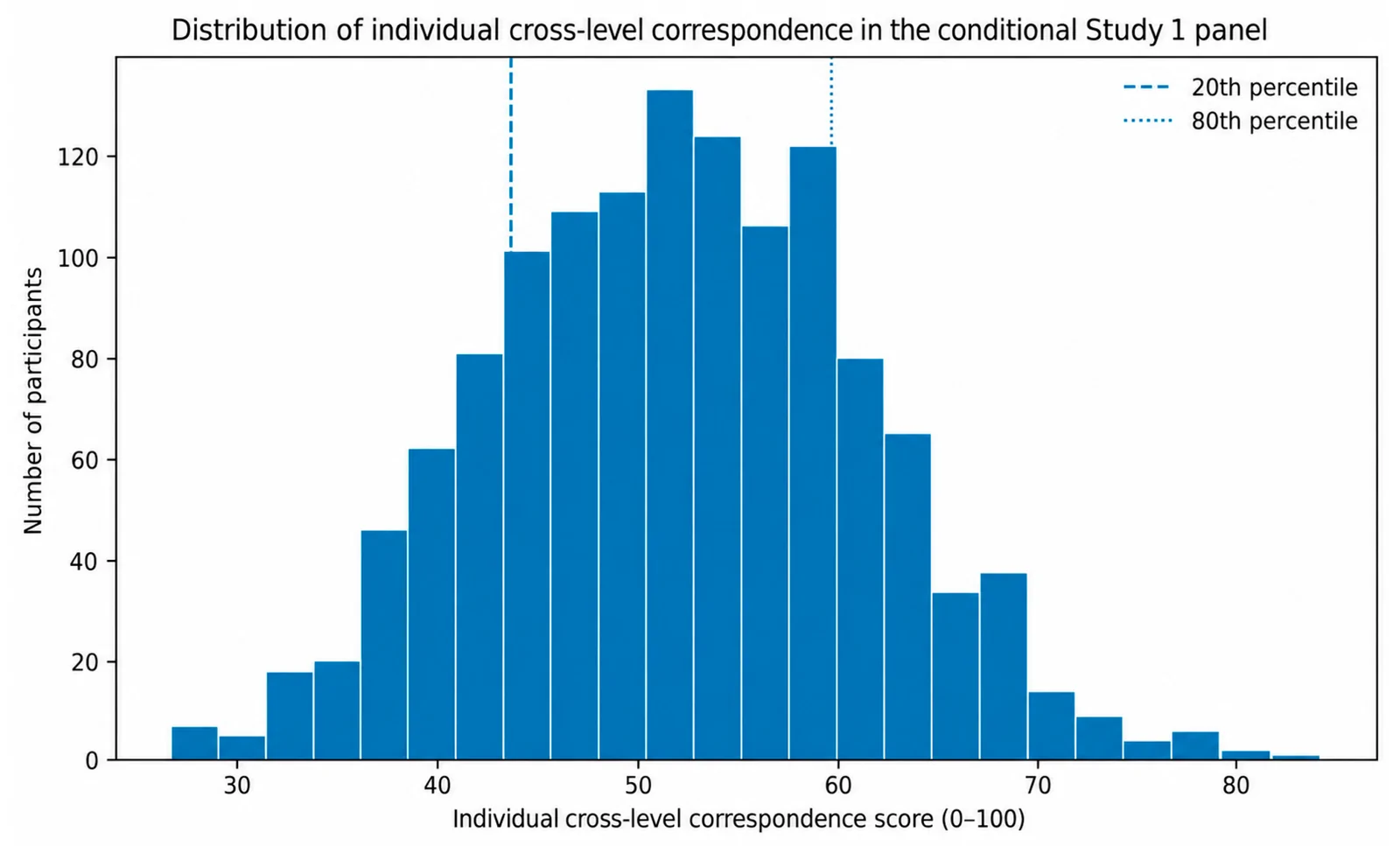
Distribution of individual cross-level correspondence in the conditional Study 1 panel. Dashed and dotted lines mark the 20th and 80th percentiles used for descriptive group comparisons, respectively.

The score varied continuously across the panel (M = 52.17, SD = 9.37, range = 26.73–84.06) and was approximately symmetric and unimodal (descriptive normality test *p* = .222). The bottom- and top-quintile cutpoints were 43.89 and 59.93, respectively; these groups were used for visualization rather than treated as natural classes. Original-participant projected trajectories (M = 51.90, lineage interval [50.98, 52.85]) and fully synthetic trajectories (M = 52.30, [51.52, 53.14]) did not differ reliably (difference = 0.40, [−0.27, 1.03]); the score was also unrelated to starting age (r = −0.035, [−0.120, 0.044]). Thus, the final panel does not support a simple age trend or a unique concentration of low correspondence at age 9 (see Figure 8).

**Figure 9:**
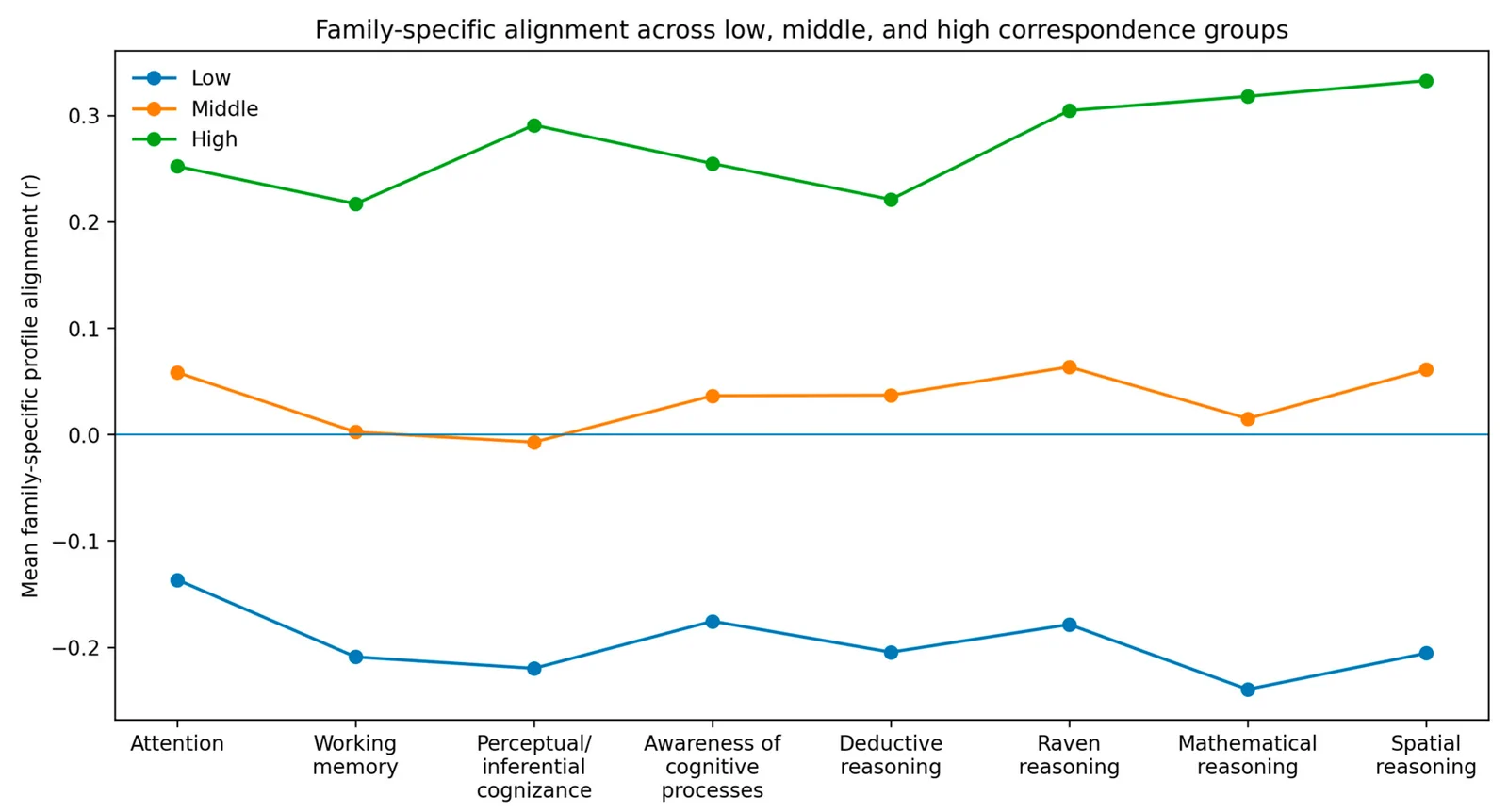
Family-specific profile alignment in the bottom, middle, and top quintiles of individual cross-level correspondence. Values show the mean agreement of each family’s relational profile with the leave-one-out age-cohort reference.

Figure 9 decomposes the score by cognitive family. The high-correspondence group showed positive alignment in every family, the middle group remained close to zero, and the low group showed negative alignment throughout. The largest descriptive high–low separations occurred in mathematical, spatial, and Raven reasoning and in perceptual/inferential cognizance, although attention, working memory, awareness of cognitive processes, and deductive reasoning also contributed. As these family-specific indices are components of the overall score, they describe where the contrast is expressed, and should not be considered independent causal predictors of correspondence.

Most importantly, individual correspondence did not behave as a stable personal trait. Scores calculated from Waves 1–5 and Waves 6–10 were essentially unrelated (r = 0.016, lineage interval [−0.058, 0.093]). An individual may therefore follow the age-normative organization closely during one segment and depart from it during another. Person-level ergodicity is better interpreted as a time-local relation between the child’s current developmental organization and the population scaffold than as a fixed characteristic or an index of developmental speed. Studies 3 and 4 tested this implication with fully observed multiwave data.

#### 3.1.4. Study 1 Conclusions

The real two-wave data show that correspondence depends jointly on cognitive process, representation level, and age. Rank stability is moderate–high in several systems, but much is age-graded. Cross-sectional and developmental change structures correspond more strongly after age adjustment, particularly at the task profile level, while remaining substantially different in magnitude. Attention is distinctive because its trajectories differentiate over the observed year. Bootstrap and whole-matrix tests support these findings, while reliability analyses qualify several change measures.

The balanced ten-wave extension conditionally produces declining long-lag rank stability, high profile preservation, raw cumulative convergence, and individualized increments; it does not add independent longitudinal evidence. Lineage sensitivity indicates that synthetic cases are somewhat smoother, and the matched-AR(1) comparison is near parity overall. The person-level analysis adds that cross-level correspondence is graded but temporally unstable; high correspondence in one segment does not define a fixed type of child or imply faster development. The strongest conclusions therefore concern the conditional consequences of the fitted model and the hypothesis that individual correspondence is phase- and interval-dependent.

### 3.2. Study 2: Developmental Dynamics, Critical Windows, and the Phase-Dependent Reorganization of Ergodicity

Study 2 involved 268 children aged approximately 4–11 years at Wave 1 and tested again one year later (Kazali et al., 2024, 2026). These children were sampled from primary schools and kindergartens in Athens, Greece, and they all came from upper-middle-class backgrounds with at least one parent having a university education. Tasks in this study addressed all processes addressed in Study 1, with an important addition of tasks addressed to relational integration. Specifically, this study addressed attention control (focusing and inhibition), working memory (word and pseudoword recall and recall of spatial arrangements), relational integration (abstraction of relations involving two, three, and four rules describing familiar animals or geometric figures), inductive reasoning addressed by Raven-like matrices, and cognizance (perceptual and inferential awareness). The extension generated Waves 3–10 for the original participants and added 532 fully synthetic participants, producing a balanced model panel of 800 children—100 per starting age from 4 through 11. Level-score reliability was generally high at Wave 1, whereas change score reliability varied across families. Detailed observed-data diagnostics, held-out prediction, model panel audits, transition threshold sensitivity, and process proxy results are reported in Supplementary S5 (Tables S5–S10).

*Variability Ergodicity:* The two observed waves showed raw one-year rank stability of 0.570 for attention, 0.636 for working memory, 0.561 for relational integration, 0.393 for Raven reasoning, and 0.227 for cognizance. Age adjustment reduced these scores to 0.139, 0.256, 0.181, 0.013, and 0.019, respectively. Adjusted Raven and cognizance stability were not distinguishable from zero, whereas the other three remained positive. Every raw-to-adjusted decline survived FDR correction (q = 0.001), and within/between variance ratios were below parity in all five families, ranging from 0.228 to 0.621.

*Structural and Dynamic Ergodicity:* Using the level-change estimator, the two-wave data yielded a profile coefficient of r = 0.736, with mean between-person correlation r = 0.531, mean change correlation r = 0.209, and MAD = 0.322. This result compares population-level structure with covariance among children’s changes, and is not a person-specific temporal covariance estimate.

Whole-matrix analysis showed age-adjusted family structural profile correspondence of r = 0.927, exact QAP q = 0.011, and MAD = 0.037; adjusted family developmental change correspondence was r = 0.827, q = 0.011, and MAD = 0.056. Task-level adjusted structural and developmental change profiles were r = 0.921 and r = 0.829, respectively, both with QAP q < 0.001. These results support strong relative profile correspondence after age adjustment, while confirming that absolute developmental relations are much weaker than population relations.

Score-level reliability was adequate for most level scores, but change score reliability was weak for working memory and only moderate for Raven reasoning. Severe empirical boundary concentration occurred in inferential awareness and selected Raven levels. Nested held-out Wave-2 prediction showed moderate performance (family r = 0.339–0.677); the transition model improved on carry-forward and fitted persistence, but did not reliably outperform the age-plus-demographic model after multiplicity correction.

*Averaging Ergodicity and SARA-C:* The observed one-year Ω ratios indicated convergence for attention (0.648), working memory (0.832), relational integration (0.765), and cognizance (0.651); Raven reasoning (0.939) did not differ reliably from parity. This pattern supports process-specific short-horizon alignment but not uniform one-year ergodicity. However, having only two waves precludes the determination of a complete cycle of construction, canalization, and renewed differentiation.

Within DPT, SARA-C is not conceived as a rigid serial chain of five processes activated one after another—it is an integrated, cumulatively expanding meaning-making mechanism in which all operations available at a given developmental level participate in cognition, while their relative developmental priority changes. Search selects potentially relevant information; Align compares it with current goals and representations and redirects further Search; Relate extracts similarities and differences from these alignments; Abstract consolidates relational outcomes into more general representations; and Cognize registers success and error, makes aspects of the process explicit when complexity requires it, and carries this information forward for subsequent use. Search and Align are therefore expected to be strongly interleaved, Relate and Abstract to overlap, and Cognize to interact with them in progressively more explicit forms.

**Figure 10:**
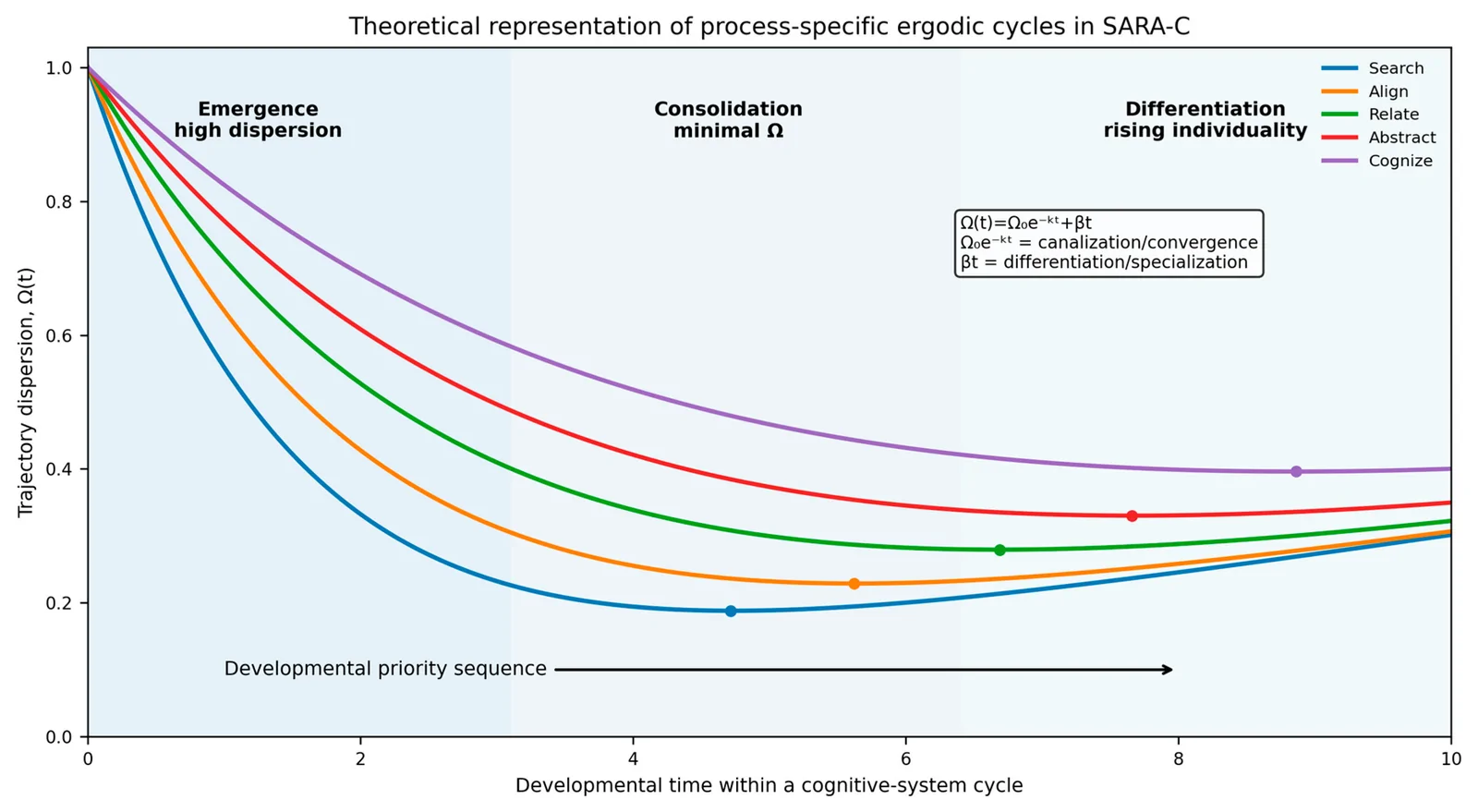
Schematic overlapping cycles of averaging ergodicity in SARA-C. Curves depict construction, consolidation, and renewed differentiation. The minimum of Ω(t) marks maximal consolidation; horizontal displacement indicates changing relative developmental priority, not a rigid serial sequence. Study 2 provides partial evidence.

Developmental priority refers to the operation undergoing the strongest acceleration, coordination, or propagation at a given period, not to the only operation active. Search–Align is expected to reorganize most rapidly in early childhood, Relate to become increasingly central in middle childhood, and Abstract and Cognize to expand from later childhood into adolescence. Perceptual awareness is expected to index an early form of Cognize, inferential awareness a later form, and awareness of cognitive processes a still more explicit form; these are overlapping probability windows rather than mutually exclusive stages. Figure 10 schematizes the construction–canalization–differentiation cycle of each operation: a minimum in Ω(t) denotes approximate maximal convergence or consolidation, whereas horizontal displacement denotes a probabilistic shift in relative priority, not a strict order of activation or completion.

To examine this formulation in the observed Wave 1–Wave 2 data, age-varying common-change loadings and change network centrality were estimated, and overlapping priority, invariant, strict-stage, and unconstrained age models were compared by held-out prediction. The mean-gain functions did not identify separate family windows: Search, Align, working memory, Relate, Abstract, and perceptual awareness reached lower-bound maxima near the youngest observed age. Relate was nevertheless the strongest carrier of coordinated change at ages 5–8 (loading = 0.758) and remained substantial at ages 7–10 (0.633); it also had the largest signed change network centrality at ages 5–8 (0.258), closely followed by working memory (0.251) and Abstract (0.242). Abstract remained strongly involved at ages 5–8 (0.694), 7–10 (0.603), and 9–12 (0.556). Search and Align became highly central in some later windows, consistent with the continued participation of earlier operations after more complex ones have emerged.

These findings support cumulative availability, overlap, and shifting relative dominance more clearly than a succession of isolated stages; they do not establish a precise timetable. Raven and relational integration task levels did not show a consistent monotonic complexity ordering. Perceptual awareness preceded inferential awareness in 77.8% of bootstrap samples, but the timing difference interval included zero. Predictive differences among the overlapping priority, invariant, strict-stage, and unconstrained models were small, so no one schedule was uniquely favored. The ten-wave model panel may illustrate the conditional consequences of asynchronous priorities, but it cannot independently validate their age ordering because all intervals after Wave 2 are generated.

Accordingly, the theoretical curves in Figure 10 should not be interpreted as empirical trajectories or as a requirement that Search, Align, Relate, Abstract, and Cognize reach their minima in a fixed serial order. The relevant predictions are that the relative contribution of these operations changes across overlapping age windows, lower-complexity forms tend to consolidate before higher-complexity forms, and increasingly explicit Cognize processes become consequential for detecting error, consolidating successful organization, and transferring it to new problems. Study 2 is compatible with this integrated, overlapping priority account, especially in showing Relate–Abstract co-organization and the continued involvement of Search–Align, but it does not uniquely establish the precise windows or the feedback role of Cognize.

**Figure 11:**
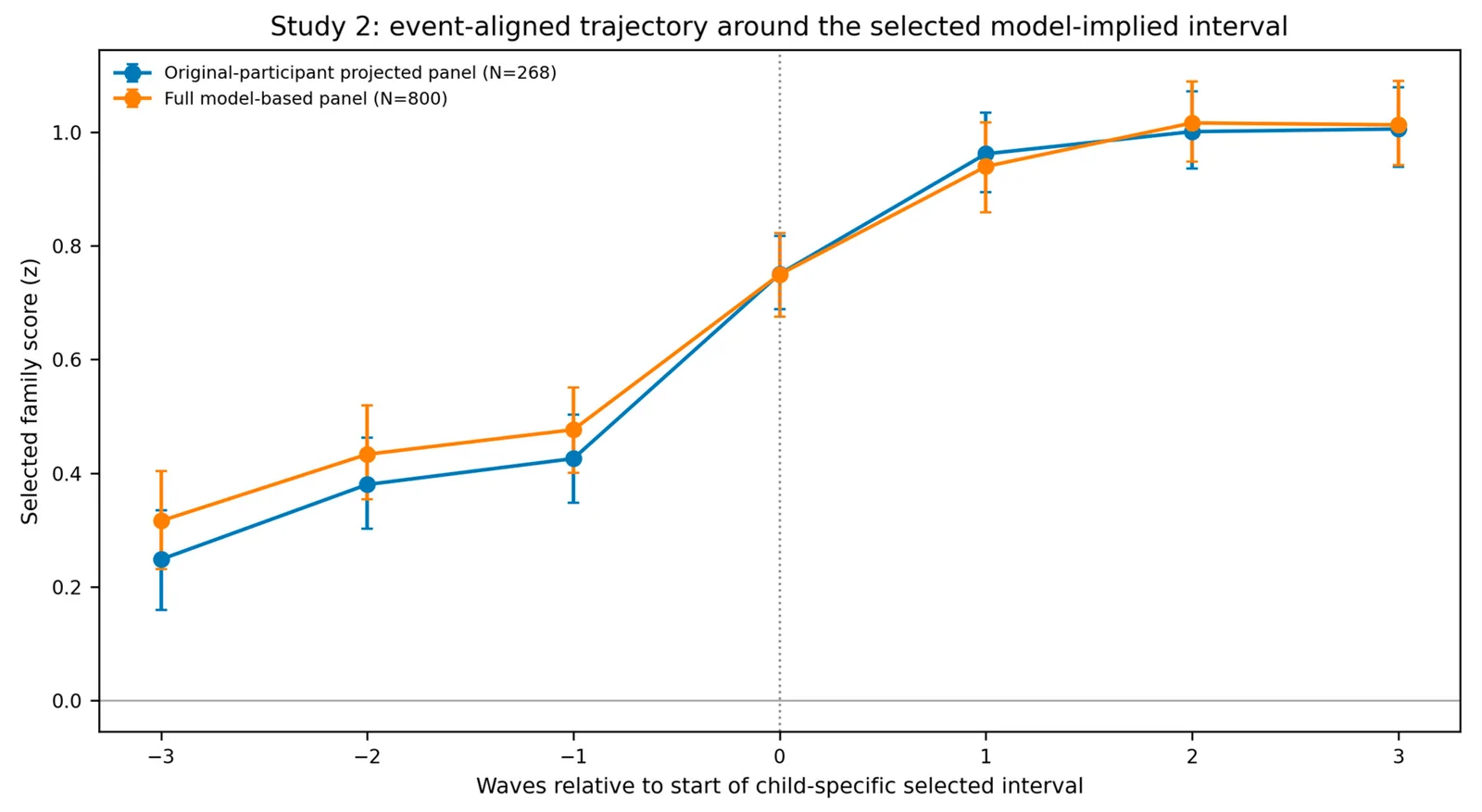
Event-aligned family-level curve in the original-participant projected panel and the full model-based panel. All intervals after Wave 2 are generated. Alignment around a selected interval reveals a localized model-implied transition that is blurred in chronological time.

*Critical Time Window:* The model-implied window was estimated as the strongest norm-adjusted adjacent-wave burst. The original-participant projected panel and the complete panel showed similar event-aligned curves, but only Waves 1–2 were observed for the original participants (see Figure 11).

The 34.3% clear-window prevalence was strongly threshold-dependent: 49.4% at z ≥ 1.50, 34.2% at z ≥ 1.75, 24.6% at z ≥ 1.96, and only 1.0% at the 95% max-search threshold. Approximately two-thirds of children did not meet the z ≥ 1.75 criterion; held-out indicators nevertheless showed partial same-family/same-interval validation, with mean held-out gain z = 0.649 and q = 0.001, albeit substantially smaller than the discovery effect. The appropriate interpretation is a model-implied local transition candidate in a subset of trajectories, not an observed developmental critical window.

#### 3.2.1. Growth Form and Regime Shifts

**Figure 12:**
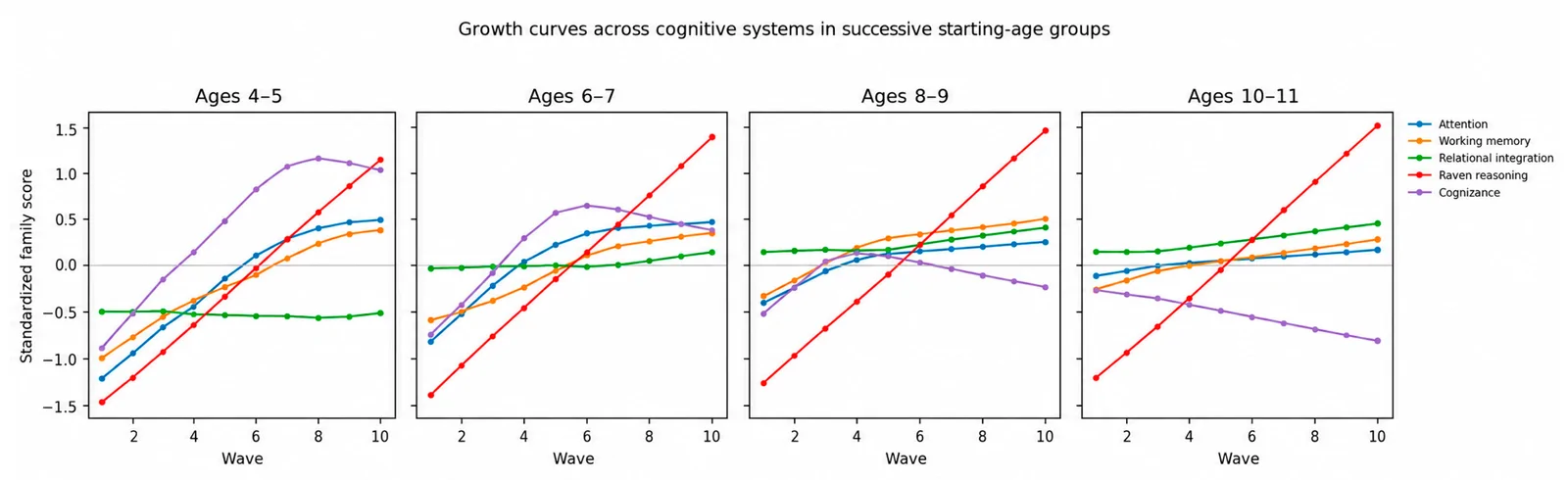
Growth curves across cognitive systems in successive starting-age groups. The curves illustrate heterogeneous growth forms across systems and ages and are conditional on the generated panel.

Several growth models were tested on the change patterns of the various processes across the 10 testing waves (Grimm et al., 2013). The Jenss–Bayley model appeared to better fit more processes than any other model, especially in the lower age windows of 4–5 and 6–7 years. In later age windows, change tended to level off as the processes approached the ceiling. Therefore, models of change are conditional on the process and the age period involved rather than on observed general developmental laws (see Figure 12).

#### 3.2.2. Process Coupling as a Function of Development

**Figure 13:**
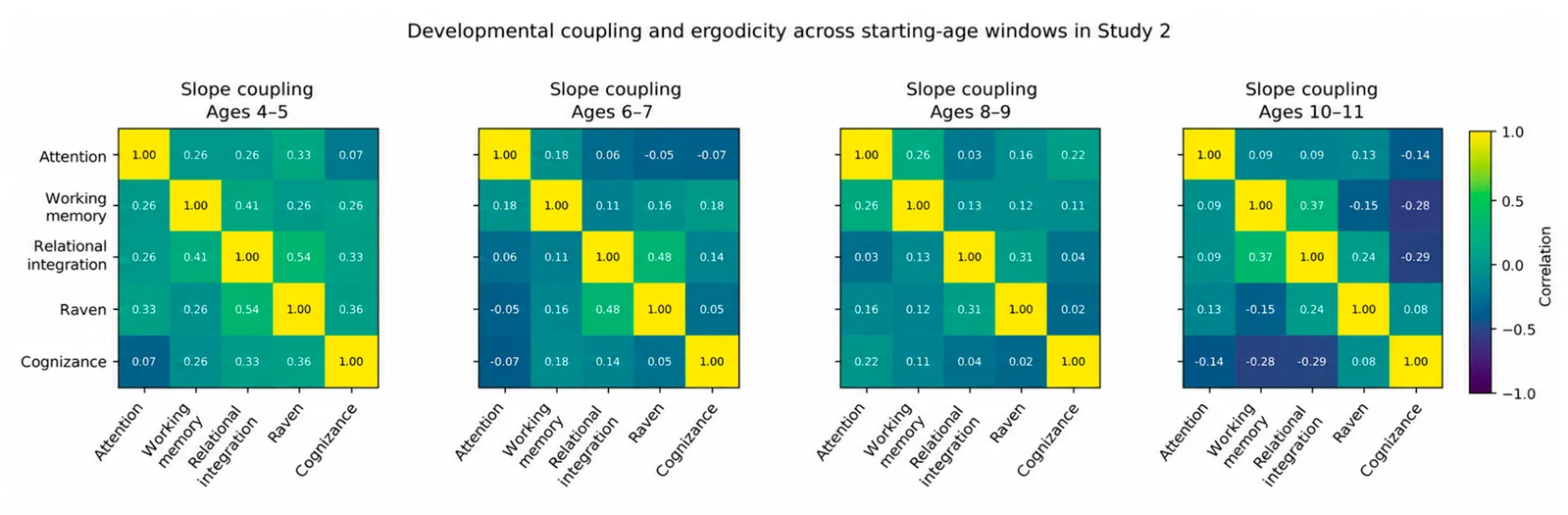
Adjusted slope correlation matrices among cognitive families for the four starting-age periods. The principal robust pattern is declining broad coupling from the youngest to later groups.

Correlations among first differences declined across starting-age periods. The mean adjusted coupling was r = 0.309 at ages 4–5, r = 0.124 at ages 6–7, r = 0.141 at ages 8–9, and r = −0.013 at ages 10–11. The youngest period exceeded each later period after FDR correction; this pattern supports a transition from broad early coupling toward later decoupling. The stronger claim that the oldest group shows selective higher-order integration is more tentative, because the overall mean was near zero and individual pairwise relations were heterogeneous (see Figure 13).

A simulation fidelity audit further showed that later generated increments remained strongly correlated with the initial observed W1-W2 increment: the mean family correlations were r = 0.918 for W2–W3, r = 0.776 for W5–W6, and r = 0.963 for W9–W10, while later increment variability compressed to ~12% of the initial level. By Wave 10, 59.6% of generated task scores lay outside the empirical W1–W2 task range, and all chronological ages were beyond the observed age support. Late-wave growth, coupling, and transition results must therefore be interpreted as extrapolative consequences of the generator.

#### 3.2.3. Study 2 Conclusions

Study 2 confirms that variability, structural, developmental change, and averaging correspondence dissociate and depend on the process and timescale. The two observed waves show process-specific rank persistence, strong adjusted relative profile correspondence, weaker absolute coupling, and short-term convergence in several systems. The model-based extension conditionally illustrates long-lag reordering, threshold-dependent local transitions, heterogeneous growth forms, and declining broad process coupling.

Two qualifications are important. First, Study 2 supports SARA-C primarily as an integrated system of cumulative, overlapping priorities rather than as a fixed sequence of process minima. Relate was the strongest carrier of common change at ages 5–8 and remained substantial at ages 7–10; Abstract participated across ages 5–12; and Search–Align remained active in later windows. However, boundary-dominated gain functions, inconsistent task complexity ordering, weak perceptual awareness versus inferential awareness timing evidence, and small predictive differences among alternative age models prevent specification of an exact timetable. Second, the model-implied transition prevalence depends on threshold and characterizes a minority at the z ≥ 1.75 criterion. The extension is informative as a generative stress test, but derivative carry-forward, compressed later increments, and extensive extrapolation require caution.

### 3.3. Study 3: Three-Wave Validation of Cross-Level Correspondence in Middle Childhood

Studies 1 and 2 establish what can be learned from two observed waves, but their change matrices remain between-child summaries and cannot test whether the same organization of change recurs across successive intervals. Study 3 provides an observed multiwave test, which included 313 Greek-speaking children from Nicosia, Cyprus, assessed annually on three occasions, beginning at 6.25–11.58 years and extending to approximately 13.58 years. Analyses were restricted to the following processes, measured empirically at all three waves: attention (perceptual focus, perceptual inhibition, and conceptual control), working memory (verbal, digit, and spatial recall), inductive reasoning (spatial and analogical), and deductive reasoning (Demetriou et al., 2008). Simulated awareness scores were excluded. The nine observed indicators were combined into four equal-weight domain scores. Detailed domain definitions, rank and variance diagnostics, three-wave structural and developmental change correspondence, factor retention, prospective prediction of Wave 3, and matched-AR(1) results can be found in Supplementary S6 (Tables S11–S17).

*Variability Ergodicity:* Age- and sex-adjusted rank stability was substantial over adjacent annual intervals but lower across the complete two-year lag. Stability ranged from 0.580 to 0.759 for Wave 1–Wave 2, from 0.785 to 0.880 for Wave 2–Wave 3, and from 0.483 to 0.691 for Wave 1–Wave 3. In every domain, the two-year coefficient was lower than the mean adjacent-interval coefficient after FDR correction (all q < 0.001). Stability was also higher during the second than the first interval, consistent with local consolidation rather than time-invariant ordering. Within-person/between-person variance ratios ranged from 0.287 to 0.584. The same lag principle appeared at the denser timescale of Study 4, where attention performance stability declined from approximately 0.65–0.71 between adjacent sessions to 0.26–0.33 across 29 sessions.

*Structural Ergodicity:* Three waves permit a pooled within-person covariance matrix based on repeated deviations around each child’s three-wave mean. At the four-domain level, profile correspondence was r = 0.774, but the exact permutation test was nonsignificant because only six relations were available (q = 0.120). At the nine-task level, profile correspondence was moderate and reliable (r = 0.454, 95% CI [0.151, 0.610], QAP q = 0.003). The mean between-person and pooled within-person correlations were 0.127 and 0.027, respectively (MAD = 0.131). The population structure therefore retained information about relative task ordering but substantially overstated within-child coupling. This result resembles the broad Sessions 1, 15, and 30 portion of Study 4 (mean between-person r = 0.386; mean within-person r = 0.027), whereas the densely repeated attention core showed stronger coupling (0.721 and 0.457). Dense repetition of a coherent trained system can reveal relations that three widely spaced observations of broader domains cannot.

*Developmental Change Correspondence:* The third wave allowed the two annual intervals to be compared directly. The mean correlations among the four residualized domain changes were 0.029 for Wave 1–Wave 2 and −0.039 for Wave 2–Wave 3. More decisively, the same children did not retain a relative tendency to change more than their peers; in particular, the correlations between residualized changes across the two intervals were −0.057 for attention, −0.004 for working memory, −0.038 for inductive reasoning, and −0.008 for deductive reasoning (all q = 0.950). The task-level profile correlation between the two change matrices was 0.220 and nonsignificant. Developmental change organization and developmental rank therefore did not automatically reproduce from one annual interval to the next.

Individual Developmental Alignment: Each child’s nine-task change vector was compared with the leave-one-out mean change vector of the corresponding starting-age cohort. The mean alignment was moderate within each interval: 0.477 for Wave 1–Wave 2 (95% CI [0.447, 0.505]), and 0.505 for Wave 2–Wave 3 ([0.480, 0.529]). However, individual alignment did not persist across intervals (r = −0.042, 95% CI [−0.161, 0.089], *p* = .463), nor did early alignment predict the child’s mean gain during the next year after controlling prior gain, baseline level, age, and sex (standardized β = −0.046, 95% CI [−0.224, 0.132], *p* = .614). Children may therefore follow the cohort-typical direction moderately well during a given year without being consistently more ergodic or developing faster in the following year.

*Psychometric Organization versus Developmental Generality:* A broad first psychometric component was retained by parallel analysis at all three waves, explaining 50.7%, 41.0%, and 36.7% of domain variance, respectively. Loading congruence was 0.988 between Waves 1 and 2, 0.986 between Waves 2 and 3, and 0.953 across the two-year lag; individual psychometric scores were also stable (r = 0.795, 0.880, and 0.711, respectively). The change dimensions behaved differently, with a first residualized change component only marginally retained for the first interval and not retained for the second; in particular, although their loading congruence was 0.820, children’s scores on the two change dimensions correlated only 0.023. A similar-looking developmental direction therefore did not identify the same children as relatively rapid developers. Stable psychometric organization and stable developmental organization are not equivalent.

*Annual Transition Architecture and Predictive Validation:* The two annual transition matrices were highly similar overall (profile r = 0.987), but the similarity was dominated by target-domain autoregression. Autoregressive paths ranged from 0.571 to 0.788 in the first interval and from 0.790 to 0.894 in the second. None of the 24 off-diagonal cross-domain paths survived FDR correction. Models fitted to Wave 1–Wave 2 were then applied prospectively to the real Wave 3. Carry-forward or target-domain autoregression predicted Wave 3 as well as or better than age-only, ordered-priority, reverse-order, and unrestricted cross-domain models.

*Averaging Ergodicity:* Three-wave cumulative dispersion declined in every domain, with Ω(3) ranging from 0.754 to 0.851. Observed-to-matched homogeneous AR(1) ratios ranged from 0.996 to 1.006, indicating no excess beyond the finite-sample autoregressive expectation. Therefore, three annual waves permit cross-interval replication and a held-out real outcome. These patterns will be further examined below in Study 4, which included 30 testing waves in some processes.

#### Study 3 Conclusions

Three annual waves are substantially more informative than two because they permit pooled repeated-measures covariance, cross-interval replication, and prediction of an observed later wave. Thus, Study 3 addressed the limitations of the two-wave designs of Studies 1 and 2. First, it showed that stable cognitive organization did not imply stable developmental momentum, as children showing greater relative change in the first annual interval were not necessarily those showing greater change in the second. Second, this study showed that the next cognitive state may be predicted mainly by domain-specific continuity rather than a broad cross-domain rule of change. Models based on each domain’s previous level predicted the third wave better than unrestricted cross-domain models.

Even three annual waves are too few and sparse to capture how change occurs during intensive learning episodes. Study 3 tested whether developmental organization persists across years but was minimally sensitive to microgenetic changes. Thus, Study 4, detailed below, aimed to examine how short-term organization unfolds throughout a 30-session training experiment and how change propagates from the trained to other, non-trained processes.

### 3.4. Study 4: Intensive Attention Training as a Function of Executive, Reasoning, and Cognizance Processes (Ages 5–7)

Study 4 examined 155 children aged approximately 5–7 years; the sample included 88 males and 67 females. Children completed 30 sessions, approximately once every two weeks, in three tasks addressed to different forms of attention control. (1) *Perceptual Inhibition Control* (PIC): In this task, children were asked to specify which of two objects in several task pairs was bigger in reality, inhibiting perceptual interference from the other object in the pair that shows it to be bigger than it really is. (2) *Conceptual Inhibition Control* (CIC): In this task, children saw pairs of numbers from 1 to 10 and had to select the larger. The task addressed the numerical distance effect—with increasing distance between the two numbers, identifying which is larger becomes easier. This task also examined inhibition of the mental bias imposed by the distance effect. (3) *Object–Name Mapping* (ONM): In this task, children saw pictures of colored objects while hearing acoustic labels naming either *the same object, another object, or a color* (e.g., a green apple with the label ‘‘apple”, ‘‘green”, ‘‘orange”, or ‘‘red”). Children have to select one of three options: an “object face” if the prompt named the object’s identity, a “color face” if the label named the object’s color, or a “cheating face” if it matched neither. Children also self-evaluated their PIC and ONM performance after every session. Furthermore, working memory, relational integration, Raven reasoning, and cognizance were addressed through the tasks used in Study 2, and language and mathematics were addressed through tasks used in Study 1. These cognitive domains were assessed at Sessions 1, 15, and 30. The design therefore permits comparison of dense temporal organization within a trained system with sparse three-wave organization across broader cognitive domains. Detailed intensive-series and three-anchor results, including P-technique and VAR benchmarks, matched-null comparisons, predictive validation, and selection-aware transition analyses, are reported in Supplementary S7 (Tables S18–S25).

**Figure 14:**
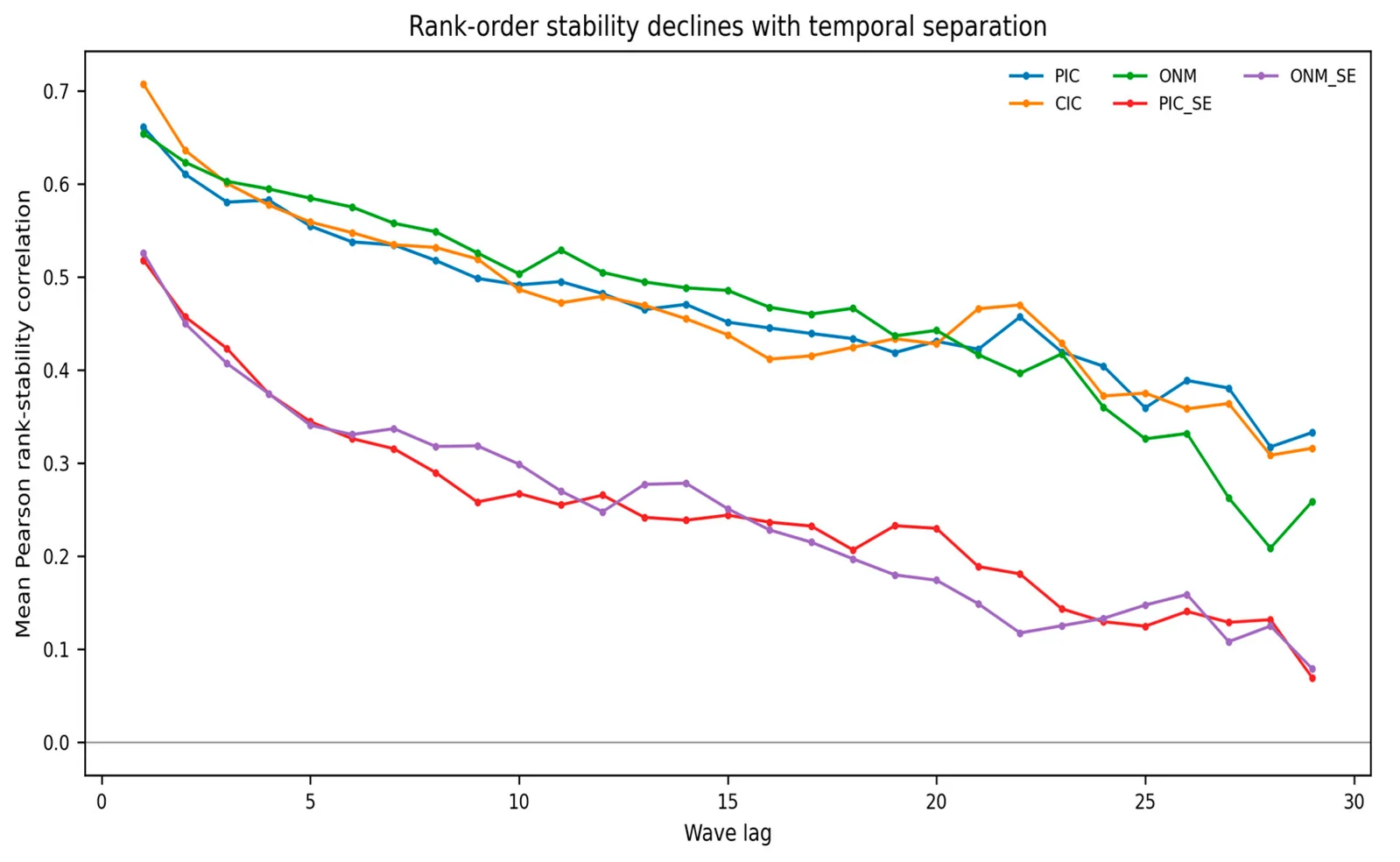
Rank-order stability as a function of temporal separation in Study 4. Performance task ordering remains locally stable but declines across the training interval; self-evaluation ordering is almost completely reorganized.

*Variability Ergodicity:* The values 0.694, 0.802, and 0.822 for PIC, CIC, and ONM, respectively, are within/between standard deviation ratios, not variance ratios; their arithmetic mean is 0.773. Lag-1 rank stability was 0.661, 0.708, and 0.654 for the performance measures, and 0.518 and 0.526 for the self-evaluations. At Lag 29, the coefficients fell to 0.333, 0.316, 0.259, 0.069, and 0.079. Every Lag-1-to-Lag-29 decline survived FDR correction (q < 0.001); local continuity therefore did not (see Figure 14).

*Structural Ergodicity:* For the three attention performance variables, the mean between-person correlation was 0.721, and conventional person-centered within-person correlation was 0.457, yielding MAD = 0.264 and profile r = 0.991. After removing common occasion effects, person-specific linear trends, and serial dependence, the mean within-person coupling fell to 0.245 and MAD increased to 0.476, while the ordering PIC-CIC > CIC-ONM > PIC-ONM was preserved.

Because the attention core profile contains only three unique edges, the near-one profile coefficient is intrinsically unstable; exact joint row-and-column permutation gave q = 0.286. The robust conclusion is preservation of relative ordering combined with substantial magnitude non-equivalence. Adding self-evaluation reduced the mean AR(1)-innovation coupling to 0.103 and increased MAD to 0.421.

*Dynamic Ergodicity:* For the attention core, the mean correlation among children’s net Session-1-to-Session-30 gains was r = 0.355, whereas the mean correlation among adjacent-session increments was r = 0.183; the corresponding five-variable means were r = 0.208 and r = 0.074. Thus, the total change across children suggested more coupling than dense temporal increments within the training series.

Formal idiographic benchmarks supported a partially shared but heterogeneous attention organization. P-technique loading congruence for the three performance tasks had a median of 0.973, with 90.3% of children above 0.85. With self-evaluation included, the median congruence fell to 0.798, and only 40% exceeded 0.85. A pooled VAR retained a weak common dynamic scaffold, but person-specific regularized VAR profiles corresponded only modestly with the pooled profile (median r = 0.277; 5.2% above 0.50). Group-level dynamics therefore represented most individual children only approximately.

*Individual Congruence and Learning Rate*: Person-specific P-technique loading congruence with the group attention pattern was high in both halves of training; the median was 0.950 during Sessions 1–15 (95% CI [0.917, 0.969]) and 0.983 during Sessions 16–30 ([0.969, 0.989]). However, the children who were most congruent early were not the same children who were most congruent later (r = −0.072, 95% CI [−0.207, 0.093], *p* = 0.372). Early congruence also failed to predict the later composite learning slope after controlling early slope, Session-15 performance, age, and gender (standardized β = −0.086, 95% CI [−0.218, 0.047], *p* = .204). High local correspondence was therefore common, but did not identify faster later learners.

**Figure 15:**
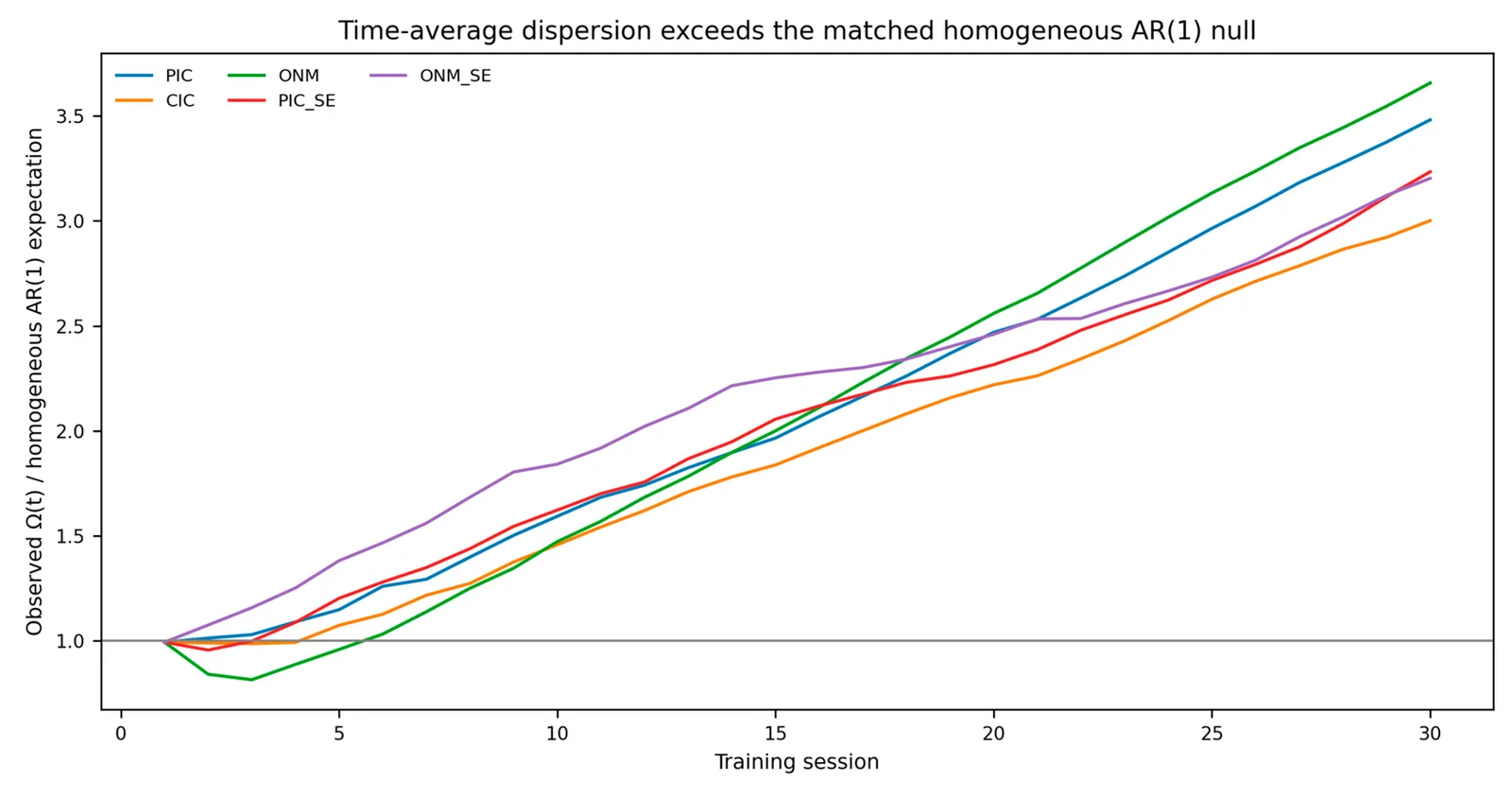
Observed time-average dispersion in Study 4 relative to the matched homogeneous AR(1) null. The comparison tests homogeneous interchangeability, not a null preserving stable child-specific levels.

*Averaging Ergodicity:* Scores were standardized separately at each session, and cumulative individual means were compared with matched finite-sample nulls. Relative to a homogeneous stationary AR(1) process, the terminal Ω(30) was 3.03–3.69 times the null expectation across the five constructs (all q < 0.001) (see Figure 15). Thus, these results are complementary to the results of Study 3: three annual waves permit cross-interval replication and a held-out real outcome, whereas 30 sessions permit analysis of local dynamics and transition timing.

A second null preserved stable between-child heterogeneity through a random intercept. Under this model, the observed/null ratios were 0.978–0.991, and none differed from parity (all q = 0.644). The result therefore rejects homogeneous interchangeability but does not require additional terminal divergence beyond stable child-specific levels.

Prospective validation fitted early-session models and predicted real Sessions 16–30. Locally updated one-step models improved prediction for PIC, CIC, and both self-evaluations after FDR correction; long-horizon recursive models were less reliable, especially for ONM. This supports local transition information but not unrestricted reconstruction of complete future trajectories.

For the domains measured only at Sessions 1, 15, and 30, relational complexity and cognizance showed the strongest internal consistency and within-person coupling. However, working memory, Raven, and especially mathematics reliability was weak or unstable. These analyses provide score-level comparability evidence, not formal longitudinal measurement invariance. All 28 possible sparse three-wave attention designs underestimated the structural discrepancy seen in the full 30-session data, demonstrating that measurement density changes what can be inferred.

#### 3.4.1. Training-Related Transition Points

The change-point analyses showed widely dispersed candidate sessions and detected clearly supported transitions in only subsets of children. Because training, repeated testing, task familiarization, motivation, and spontaneous development cannot be separated without a comparison condition, these events are described as training-related transition points rather than critical developmental windows.

The same exhaustive search was calibrated against child-specific smooth-learning AR(1) null trajectories. The proportions exceeding the child-specific 95% null threshold were 13.5% for PIC, 18.1% for CIC, 9.0% for ONM, 20.6% for PIC self-evaluation, and 20.0% for ONM self-evaluation; all five survived FDR correction. At the 99% threshold, the rates fell to 1.9–5.2%. Performance transitions occurred about three sessions later than self-evaluation transitions. Timing correspondence was selective within PIC-CIC and within the two self-evaluations, not governed by one domain-general timing factor.

Event alignment was also compared with a null that underwent the same event selection procedure. PIC and both self-evaluation measures showed aligned peaks exceeding selection alone; CIC did not, and ONM was marginal. Across 64 models testing transfer to untrained processes, no coefficient survived FDR correction. The data therefore support heterogeneous local reorganization during training, not a universal transition or an automatic global cascade.

ONM illustrates why rank stabilization and trajectory convergence are distinct. Rank stability increased from 0.575 early in training to 0.716 later, while trajectory dispersion also increased from 0.549 to 0.689; both changes had q = 0.005. Children became more consistently ordered without becoming more alike.

#### 3.4.2. Study 4 Conclusions

A narrowly trained system can show strong local organization without being globally ergodic. The three attention processes formed a recognizable common organization, but within-person coupling was substantially weaker than between-person coupling, rank correspondence declined with temporal distance, and pooled dynamics represented most individual children only approximately. Thus, dense measurement revealed local organization that sparse assessment would have overstated. Performance and self-evaluation were associated at the group level, but their moment-to-moment coordination was weak. Rank correspondence declined with temporal distance, pooled dynamics generalized only partially to individuals, and homogeneous time-averaging failed because children retained stable individual differences. Individual loading congruence was high locally, but neither stable across training halves nor predictive of faster later learning.

Developmental transitions were heterogeneous and did not automatically generalize. Only subsets of children showed transition points exceeding random change, reflecting the fact that transition timing differed across processes and children. Rank ordering of individuals appeared to become more stable, but trajectories became more dispersed, and local attention learning did not reliably transfer to broad cognitive change. Children may therefore become more consistently different without becoming more developmentally alike. Therefore, sparse measurement may overstate structural correspondence because rank stabilization can coexist with increasing trajectory dispersion.

### 3.5. Psychometric g, Developmental Generality, and SARA-C: From Cognitive State to Developmental Priority

The ergodicity analyses raise a direct question for theories of intelligence: can the general factor observed across persons at one occasion be treated as the mechanism that produces intellectual change within persons? The findings require three related but distinct constructs. Psychometric g, *gP*(t), is the main common dimension of attained cognitive levels at time t. Interval *developmental generality*, *g*Δ(t1,t2), is the common covariance among age- and prior-level-adjusted changes over a specified interval. Developmental g in the stronger mechanistic sense, *gD*,*i*(t), would be the person- and phase-dependent efficiency with which cognitive reorganization is initiated, coordinated, consolidated, propagated, and transferred. A factor extracted from one interval of change is therefore an empirical projection of this operator, not necessarily the operator itself. The operational definitions, score construction, factor extraction and retention procedures, and supporting analyses of psychometric g, interval developmental generality, dense learning generality, and the candidate developmental operator are detailed in Supplementary S9 (Tables S32–S36; Figure S3).

#### 3.5.1. Generality Is Stronger in Cognitive States than in Developmental Transitions

A broad *psychometric g* was recovered at every real testing wave. In Study 1, it explained 58.4% and 51.9% of domain-level variance at Waves 1 and 2, respectively, and individual g scores correlated 0.891 across waves. In Study 2, it explained 66.7% and 59.3%, with score stability of 0.740. In Study 3, it accounted for 62.1%, 52.7%, and 46.1% across three real waves, with a two-year score correlation of 0.809. Study 4 yielded the same basic pattern during training: *psychometric g* explained 35.3%, 41.7%, and 38.8% at Sessions 1, 15, and 30, respectively; loading configurations were highly congruent; and score correlations ranged from 0.772 to 0.811. Psychometric g is therefore a robust coordinate of generalized cognitive organization already attained.

The organization of change was consistently weaker and less stable. Study 2 produced the clearest one-year developmental dimension: *g*Δ explained 41.0% of residualized change variance and was centered on relational integration, working memory, and Raven reasoning. Study 1 yielded a weaker dimension, explaining 22.4%, centered on mathematical, spatial, and Raven reasoning. In Study 3, the first interval supplied only marginal evidence for common change, the second supplied none, and developmental scores from successive annual intervals were essentially unrelated. Study 4 again showed phase dependence: the first interval dimension explained 25.5% and was retained, whereas the second explained 22.3% but required two components; the two interval scores correlated only 0.137 (95% CI [−0.065, 0.267]). Thus, the positive manifold among attained levels does not reappear as an equally broad or temporally stable manifold of change.

Initial *psychometric g* was nearly unrelated to relative multivariate advancement in Studies 1–3: the correlations were 0.117, 0.045, and 0.074, respectively. This does not mean that higher-g children fail to improve or that present g is useless for predicting later standing. Because cognitive levels are stable, *gP*(t) usually predicts *gP*(t + 1). The narrower conclusion is that current standing is a poor proxy for residual developmental momentum—who advances more or less than expected from age and starting level.

**Figure 16:**
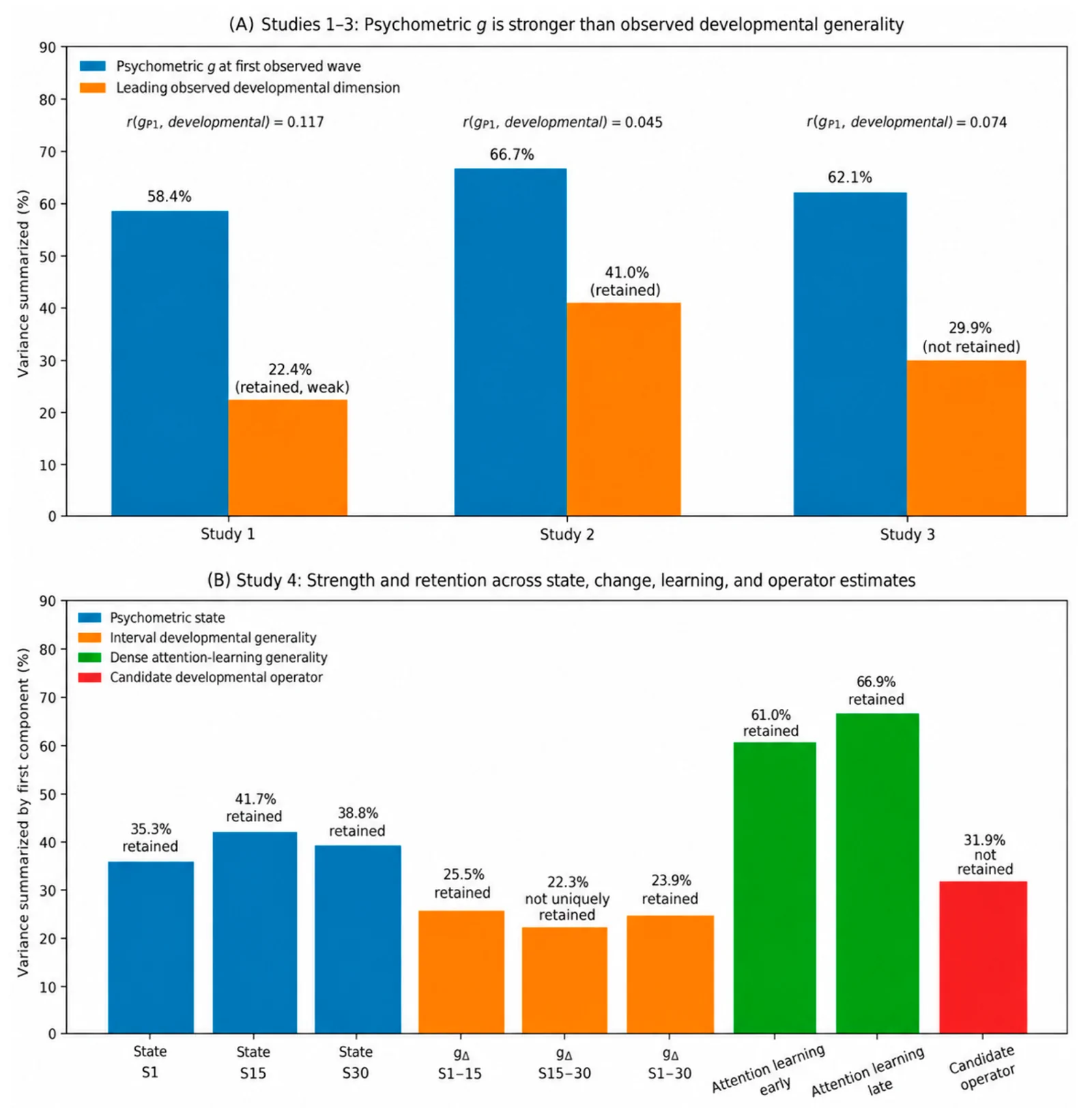
Strength of psychometric state and developmental generality across Studies 1–4: (**A**) Psychometric g at the first observed wave and the leading observed developmental dimension in Studies 1–3; annotations show the correlations between initial psychometric g and the principal developmental estimate. (**B**) Strength and retention of psychometric state, interval developmental generality, dense attention learning generality, and the candidate developmental operator in Study 4. All values are based on real participants and observed waves or sessions. Retention refers to Horn parallel analysis; the second Study 4 interval required more than one dimension, and the candidate developmental operator was not retained.

Study 4 adds an important qualification: Psychometric g predicted some breadth of cross-domain reorganization during structured training. In particular, the standardized coefficients were 0.360 for Sessions 1–15 and 0.333 for Sessions 15–30. These relations were not uniform across domains and were attenuated when the analysis was restricted to the more reliable measures. More decisively, *psychometric g* did not predict early or later learning rates in the densely measured attention processes. Psychometric g may therefore constrain how broadly change is expressed under favorable learning conditions, but does not by itself identify who will learn a particular process fastest. Figure 16 summarizes this contrast across all four studies, preserving the observed Studies 1–3 comparison and adding the psychometric state, interval change, dense learning, and candidate operator estimates from Study 4.

#### 3.5.2. Developmental Generality and the Construction of the Next Psychometric State

The loading pattern of *g*Δ identifies the processes through which reorganization is routed during a particular interval; no single process occupied this role universally. Study 2 was Relate-centered, Study 1 was centered on higher-order representational and reasoning systems, and Study 3 was attention-centered without yielding a stable broad developmental factor. In Study 4, relational integration, language, Raven reasoning, and attention carried the first interval, whereas later change separated into a working memory–relational integration–Raven route and an attention–language route. Developmental generality is therefore better understood as a vector of coordinated change than as an undifferentiated increase in one quantity.

Study 4 directly tested whether such a vector contributes to the next psychometric state. Controlling Session-1 g, first-interval *developmental generality* predicted Session-30 g positively (β = 0.263, 95% CI [0.159, 0.367]). Thus, broader early reorganization contributed to later standing beyond the initial state. Conditional on the already attained Session-15 state, however, the coefficient reversed (β = −0.423, 95% CI [−0.563, −0.283]), and early *g*Δ predicted less subsequent *developmental generality*. These coefficients answer different questions. Relative to the starting point, broad early change raises the later state while, among children at the same intermediate state, those who had already reorganized more had less change remaining. Repeated cross-validation showed that *g*Δ improved the prediction of Session-30 g beyond either Session-1 or Session-15 g alone. Developmental generality therefore carries information about both accumulated advance and current phase position, but should not be interpreted as a fixed personal growth capacity.

This state transition relation may be represented schematically as follows:

x_i_(t + 1) = x_i_(t) + η_i_(t)R_p_(t)F_sara−_c[x_i_(t), e_i_(t)] + ε_i_(t),

where x_i_(t) is the current vector of cognitive levels, F_sara−_c is the integrated meaning-making operation applied to the current representation and evidence, R_p_(t) routes this operation through the processes with developmental priority, η_i_(t) represents the efficiency and reach of reorganization, and ε_i_(t) represents domain-specific, contextual, and stochastic influences. Psychometric *gP*(t + 1) is the main common coordinate of the resulting cognitive state, whereas *g*Δ summarizes the coordinated part of the transition that produced it.

#### 3.5.3. SARA-C as an Integrated, Overlapping Developmental Priority System

The findings also clarify the nature of SARA-C. It is not a rigid chain in which one process is completed before the next begins, but an integrated, cumulatively expanding meaning-making mechanism in which all operations available at a given developmental level participate in cognitive activity. Search generates potentially relevant information or strategies; Align compares them with goals, prior representations, and evidence and redirects further Search. Relations detected during Search and Align support choices and the construction of more general representations; in turn, emerging abstractions guide the detection and reorganization of further relations. Cognize registers success and error, makes representations increasingly explicit, and carries useful organizations forward into later cycles. Developmental priority concerns which operation or coupling is undergoing the greatest acceleration, coordination, or propagation, not which operation is exclusively active.

The empirical support is strongest for this broad, overlapping architecture and weaker for a precise timetable. Study 1 showed closely overlapping early-childhood maxima for Search and Align indicators, at approximately 6.5 and 6.8 years, respectively, and both dominated the early common-change profile. Study 4 likewise showed rapid early attention learning followed by deceleration, increasing rank persistence, and overlapping transition timing, especially for the two inhibition control measures. These findings support a Search–Align priority in early childhood without requiring a strict Search-then-Align sequence.

Relate received partial support as a later priority. In Study 2, relational integration was the strongest carrier of *developmental generality* and remained central across the 5–10-year windows, accompanied by working memory and Raven reasoning. Abstract development was supported more clearly by complexity-sensitive shifts: increasingly complex Raven and mathematical levels tended to reach their strongest change later in Study 1, and higher relational-complexity levels became relatively more active later in Study 4. Study 3 showed a qualitative shift from attention toward inductive and deductive reasoning, although uncertainty was wide and no broad common-change factor was retained. Together, these results support overlap between Relate and Abstract, rather than sharply separated stages.

The evidence concerning Cognize is weaker. Study 4 showed that perceptual awareness was more closely related to attention than inferential awareness at the first assessment, consistent with an early perceptual form of cognizance; however, the proposed progression from perceptual awareness to inferential awareness and then awareness of cognitive processes was not recovered consistently across studies. Self-evaluation did not prospectively predict performance correction or broad transfer, and a candidate operator combining update, monitoring, consolidation, and transfer was not retained as one factor. Cognize may therefore be differentiated by form and recruited especially around error, explicitization, consolidation, or transfer, but its feedback function was not demonstrated by the available proxies.

The prespecified overlapping priority models also did not consistently outperform invariant, strict-stage, or unconstrained alternatives in held-out prediction. The evidence consequently supports SARA-C as a cumulative and probabilistic framework, not as a uniquely established causal architecture or a universal sequence of exact age boundaries. A strong claim of developmental priority should require convergence of at least two indicators—accelerated gain, coordination of change, propagation to other systems, or predictive reach—and should not depend on reliability problems, score bounds, or practice effects.

#### 3.5.4. A Common Psychometric–Developmental Theory of Intelligence

The resulting theory distinguishes state, transition, and developmental operator. Psychometric g is reflective synchronically, as several abilities may express a common organization at a given occasion. Historically, however, this organization may be formative or emergent, accumulating through many local, overlapping, and phase-specific SARA-C cycles. Interval *developmental generality* measures the temporary breadth with which one such cycle propagates across processes. Developmental g, if the term is retained for the stronger construct, is the changing efficiency with which the system updates, coordinates, consolidates, and transfers its organization; the present studies do not support treating it as one stable scalar factor.

This account reconciles broad psychometric unity with developmental non-ergodicity. The attained state can be strongly general even when the next increment of change is selective, when different processes carry reorganization in different periods, and when different children reach corresponding transitions at different times. Stable psychometric loadings therefore do not establish stable developmental causes. Tests intended to predict later standing may rely heavily on *psychometric g* and autoregressive continuity. Tests intended to predict developmental potential must also identify the current priority, the organization of change, the efficiency of learning, and whether local reorganization consolidates and transfers. In this sense, *psychometric g* is accumulated cognitive organization, *developmental generality* is the temporary breadth of coordinated change, and SARA-C—routed according to developmental priorities—is a candidate mechanism through which one cognitive state is transformed into the next.

## 4. Discussion

### 4.1. Structured Non-Ergodicity Across Developmental Scales

The four studies examined whether the organization of differences among individuals adequately represents the processes by which individuals change. Table 3 summarizes the main findings across the four studies. Study 1 covered the broadest age span and process range; Study 2 focused on relational integration, cognizance, and domain-specificity in development; Study 3 tested cross-interval replication and prediction across three fully observed annual waves; and Study 4 observed attention learning intensively when it is a developmental priority, along with its transfer to other processes. Taken together, these studies show that cognitive development is neither globally ergodic nor random; it is better characterized as structured, selective, and phase-dependent non-ergodicity.

Across designs, relative profiles were generally more stable than intertwined individual trajectories, adjacent profiles were more stable than distant ones, and cumulative levels behaved differently from local increments. Two-wave results are short-horizon cross-level tests; generated panels describe conditional dependencies rather than independent replications; three real waves show that relative change may fail to reproduce across annual intervals; and training-related transition points cannot be identified uniquely as developmental critical windows.

The findings do not support a single coefficient that can be assigned independently of temporal interval, developmental phase, task complexity, measurement quality, and statistical decomposition, nor do they support a fixed person-level type. A process may preserve relative ordering without converging in developmental history, two matrices may have similar profiles while differing greatly in coupling magnitude, and an individual may resemble the population organization during one interval but not during another.

### 4.2. Four Dissociations in Developmental Ergodicity

The first dissociation concerns local versus long-term stability. Adjacent observations were more stable than widely separated observations. In Study 1, much raw stability was attributable to normative age ordering. In Study 2, the generated panel showed high local continuity but long-lag reordering. Study 3 reproduced the same lag dependence across annual observations, and Study 4 showed substantial decline across the 30-session interval, especially for self-evaluation.

The second dissociation concerns profile correspondence versus magnitude equivalence. Whole-matrix bootstrap and permutation procedures confirmed several adjusted profile correspondences, especially at the task level, but within-person or developmental change coupling was almost always weaker than between-person coupling. Study 3 made this especially clear with three real waves: task profile correspondence was significant, yet the mean pooled within-person correlation was 0.027, compared with 0.127 between persons. High profile correlations therefore do not establish full structural ergodicity.

The third dissociation concerns level convergence versus increment heterogeneity. Cumulative averages can converge while the sequence of gains remains individualized. The conditional Study 1 panel displayed raw convergence but little systematic excess over a matched AR(1) expectation after detrending. Study 3 showed cumulative dispersion close to its matched expectation, while individual change rankings failed to persist. Study 4 exceeded a homogeneous AR(1) null but not a random-intercept null preserving stable child differences.

The fourth dissociation concerns rank stabilization versus trajectory convergence. ONM showed that rank ordering may become more reliable while trajectories become more dispersed. Study 3 adds a related distinction: stable psychometric rank and loading structure can coexist with virtually no continuity in who changes the most from one annual interval to the next. Variability, averaging, and developmental change diagnostics are therefore not interchangeable.

The person-level analyses extend all four dissociations. In Study 1, individual cross-level correspondence varied continuously but had negligible stability across the first and second halves of the conditional panel. In Study 3, moderate alignment with the cohort-typical change vector within each year did not persist into the next year and did not predict later gain. In Study 4, high local loading congruence did not persist across training halves or identify faster later learners. Correspondence with a population scaffold is therefore a time-local property of the child–system relation, not a fixed trait or a synonym for developmental speed.

### 4.3. Ergodicity Is Phase-Dependent Rather than Hierarchically Fixed

The results do not support a simple hierarchy in which lower-level processes are always more ergodic. Attention was strongly organized across children but changed with lag and showed weak innovation-level coupling. In Study 2, relational integration and cognizance sometimes showed comparatively strong three-anchor organization during the 5–7-year period, although several other domains were limited by weak measurement reliability. Study 3 showed stronger rank persistence during the second annual interval, consistent with local consolidation, but no reliable cross-domain propagation. The temporal instability of individual correspondence further indicates that ergodicity depends on the current phase of the person–system relation, rather than on a permanent position in a hierarchy.

During emergence, alternative organizations and individual timing differences may reduce correspondence, consolidation may temporarily increase constraint, and later differentiation may again diversify pathways. The generative panels illustrate this possibility, but their exact phase sequences are model-dependent.

### 4.4. Developmental Priorities and Critical Windows

The four studies provided different kinds of temporal evidence: In Study 1, we estimated age-specific maxima in baseline-adjusted one-year gain. In Study 2, we generated threshold-dependent local-transition candidates after Wave 2. In Study 3, we used three annual waves to test whether developmental change organization and individual alignment reproduce across intervals; however, this study was not dense enough to localize transition points. In Study 4, we directly observed heterogeneous training-related transitions, although we admittedly could not separate the possible effects of training, repeated testing, and familiarization. These forms of evidence should not be combined under one unqualified label of developmental critical windows.

Phase desynchronization remains a plausible source of non-ergodicity, as children may reach local reorganizations at different times. However, developmental degeneracy provides a complementary explanation: different cognitive or neural configurations may yield similar psychometric outcomes (Di Plinio et al., 2024). Population similarity therefore need not imply identical individual mechanisms.

### 4.5. Growth Regimes and Process Coupling

Study 2 indicated that growth form and process coupling vary across starting-age periods. The broad conclusion of heterogeneous growth regimes survived formal comparison, although exact model assignments appeared to be generator-dependent and late waves tended to extrapolate. The strongest process coupling result was the declining broad coordination from ages 4–5 to later groups; thus, selective higher-order integration in the oldest group remains tentative.

### 4.6. Implications for General Intelligence and Development

The findings are compatible with Developmental Priority Theory and SARA-C, taken to operate as an integrated, cumulatively expanding architecture in which Search, Align, Relate, Abstract, and Cognize remain jointly available but operational focus changes across overlapping age windows. Admittedly, the findings did not uniquely identify SARA-C as the sole source of psychometric g; however, they showed the relative importance of these processes as carriers of change in development. Study 2 supported Relate–Abstract co-organization as the main change carrier, with continued Search–Align involvement, although the exact age schedule was rather blurred. Study 1 showed the emerging prioritization of deductive reasoning in late childhood and adolescence. Notably, however, prediction of the third wave from earlier waves in Study 3 showed no advantage for a fixed ordered-priority model over simpler domain-specific persistence; person-level correspondence also did not predict faster subsequent development. Study 4 showed that attention control and related awareness, however important they are in early and middle childhood, are not the direct catalyzers of change in reasoning processes lying higher in the mental hierarchy.

Therefore, the four studies converge on the following general postulates about the developing mind:

1. At every developmental period, cognitive processes form an organized system rather than a collection of independent abilities. Psychometric g is the principal common coordinate of this attained organization—it is powerful and broad, relatively stable, and predictive of later cognitive standing.
2. However, psychometric g describes where the cognitive system currently is, not necessarily how it will change in the following period. Hence, processes that correlate strongly across individuals at a given time need not change together within the same person. Cross-sectional population structure is therefore a developmental scaffold, not a direct roadmap of individual change.
3. Ergodicity is selective, graded, and conditional. Development is neither globally ergodic nor globally non-ergodic. Cross-level correspondence varies with process, age, developmental phase, temporal lag, measurement density, context, and person. Ergodicity is weaker during emergence, rapid reorganization, differentiation, or individualized specialization and becomes stronger when changes within children are canalized towards a shared organization. At these convergence phases, adjacent observations may correspond closely even when long-term trajectories diverge.
4. Development is cumulative over recurring cycles. Development adds new forms of control to earlier ones without replacing them. A central core, involving Search, Align, Relate, Abstract, and Cognize processes (SARA-C), operates as an integrated and recurrent meaning-making system. Over short-term learning experiences or long-term reflectively guided mental restructuring, Search to extract information, Align to enhance the scope of selection, Relate to extract rules and concepts underlying encounters, Abstract to consolidate representations, and Cognize to optimize future operation become increasingly efficient, with earlier operations remaining available if needed. Development is therefore cumulative and hierarchical, but not rigidly serial.
5. Developmental priorities shift when a certain minimum of efficiency regarding a given component is met. When a priority dominates in a period, the processes involved become the principal carriers of change, although the remaining processes continue to operate. Search and Align, expressed in the control of attention, dominate as a priority in early childhood; Relate dominates in middle childhood; increasingly complex Abstract and Cognize processes become prominent later. These windows overlap, and chronological age changes their probability rather than determining them mechanically.
6. The development of a new organization tends to pass through three recurrent conditions: construction→canalization→differentiation. Construction produces exploration, instability, and heterogeneous timing; canalization stabilizes an effective organization and may increase cross-level correspondence; differentiation allows the consolidated organization to support increasingly varied strategies, interests, and applications.
7. Architecture and change are not mutually exclusive or constrained. A common cognitive architecture can be reached through alternative developmental pathways, and similar starting states may lead to different later organizations. Individual conformity to the population pattern is therefore local and phase-dependent rather than a fixed personal trait. Thus, children may become more stably ordered in recurring cycles while their developmental trajectories simultaneously become more dispersed.
8. Generality must be distinguished at three levels: *Psychometric* $g_{P}\left(t\right)$ represents the common organization already attained at a particular occasion. *Interval developmental generality* $g_{\Delta }\left(t_{1},t_{2}\right)$ represents the temporary breadth and direction with which processes change together during a particular interval. *Developmental* $g_{D}\left(i,t\right)$, represents the person- and phase-specific efficiency with which the system updates, coordinates, consolidates, propagates, and transfers its organization.

### 4.7. Methodological Implications

Cross-sectional analyses are most informative when cross-level correspondence has been demonstrated for the process, age, and timescale concerned. Two-wave designs identify stability, dispersion, and covariance among individual differences in change, but not whether the same change organization recurs. Three observed waves, as in Study 3, permit pooled repeated-measures covariance, comparison of two successive intervals, and prediction of a real later wave, but still cannot support reliable person-specific temporal models or localize transition points. Intensive designs, as in Study 4, reveal local organization but may not show whether changes consolidate over years. Accelerated multiwave and multiscale designs remain especially valuable.

The P-technique and VAR benchmarks in Study 4 show that the present diagnostics complement rather than replace formal idiographic methods. A common attention loading pattern was evident, yet broader performance–self-evaluation organization and person-specific dynamics were heterogeneous. DSEM or GIMME would require longer or denser series than Studies 1 and 2 provide and would be fragile, with only 30 occasions and 5 variables without strong regularization.

Measurement density changes the recovered estimand. Study 3 and the three-wave portion of Study 4 both yielded very weak average within-person coupling across broad cognitive systems, whereas the 30-session attention core showed substantially stronger coupling within a narrow trained system. Sparse three-wave subsamples of the intensive series also made cross-level structures appear more similar than the complete 30-session analysis. Person-level indices require the same caution; in particular, their stability and predictive value must be tested on temporally distinct observations, rather than inferred from a score computed over the same period.

### 4.8. The Role of Simulation

The model-based extensions ask what longer-term patterns would follow if calibrated age functions, one-year transitions, covariance structures, and phase shifts continued to operate; they are not independent longitudinal replications. Synthetic participants do not increase empirical inferential precision, and the selected-panel intervals are conditional on donor lineages rather than uncertainty across all model classes, refits, seeds, and hyperparameters.

The extensions remain scientifically useful as generative stress tests. They show how local stability can coexist with long-term reordering and how profile correspondence can coexist with weak magnitude coupling. The audits also show that synthetic interpolation can smooth trajectories, later increments may remain strongly tied to the initial derivative, and late waves may leave observed age and score support. Study 3 provides an observed benchmark; namely, broad cross-domain priority paths fitted to one annual transition did not improve prediction of the independently observed third wave. The individual Study 1 score likewise remains a conditional model result until it is reproduced in longer observed panels.

### 4.9. Limitations and Future Directions

Studies 1 and 2 had only two observed waves and model-implied longer trajectories; Study 3 partially addresses this limitation, with three fully observed annual waves, two real developmental intervals, and a held-out real outcome; and Study 4 provides dense repeated measurement. However, three annual waves still do not support reliable person-specific covariance models, and 30 training sessions do not establish long-term developmental stability. Designs combining at least three annual waves with denser measurement around predicted transitions are needed.

Measurement comparability is a second limitation. Composite-level reliability and score boundary diagnostics showed weak change reliability and floor/ceiling concentration in several domains. Item-level longitudinal invariance and differential item functioning analyses were not possible from the archived files. Apparent covariance change may therefore reflect both developmental reorganization and changing measurement.

Third, Study 4 lacked an untrained comparison condition. Training-related learning, repeated testing, task familiarization, motivation, and maturation cannot be separated. Transition points are consequently interpreted as local training-related reorganizations, not developmental critical periods in a causal sense.

Fourth, the samples were drawn from a restricted Greek-speaking cultural context and were not designed to establish universal developmental laws. Cross-cultural, linguistic, socioeconomic, and educational replication is required. Finally, the individual Study 1 score depends on a selected generated panel and on only ten observations per person; as such, its negligible split-period stability may reflect genuine phase dependence, estimation noise, or both. The observed Study 3 and Study 4 analyses reduce this uncertainty, but do not eliminate the need for longer person-specific series.

## 5. Conclusions

In short, the present findings point toward a common theory of developing intelligence that integrates classical and contemporary psychometric approaches (Carroll, 1993; Jensen, 1998; Kovacs & Conway, 2016; Spearman, 1927; van der Maas et al., 2017) with major developmental theories (Case, 1992; Halford et al., 1998; Pascual-Leone & Johnson, 2021; Piaget, 1970; Siegler, 1996). The central implication is that theories of attained cognitive organization and theories of developmental change describe different—albeit reciprocally related—levels of the same system.

For psychometric theory, the findings confirm that *g* is a robust coordinate of attained cognitive organization, but they show that it is not by itself the factor organizing change within individuals. Spearman, Carroll, and Jensen would therefore need to distinguish the generality of cognitive state from the generality of developmental transition. Mutualism would need to become a time-varying, person-specific, and phase-routed network model, because interactions among processes were neither constant nor equally general across development. Process overlap theory would likewise need to treat process overlap as developmentally variable. Attention and executive control were not universal carriers of change, and relational integration and higher-order reasoning became the principal bottlenecks or relays during other developmental phases.

For developmental theory, the findings show that a coherent structure observed across children is not necessarily the structure governing change within each child. Piagetian stages would therefore be better conceived as probabilistic developmental attractors than as homogeneous states traversed in the same way by all children. Processes have overlapping developmental windows, children enter periods of reorganization at different times, and the principal carrier of change shifts across attention, relational integration, and higher-order reasoning. Neo-Piagetian parameters such as Pascual-Leone’s *M*-power, Case’s working memory capacity, and Halford’s relational complexity limit remain important constraints, but no single age-indexed parameter is sufficient to explain the observed organization of change. Siegler’s overlapping strategies capture local variability and microgenetic transition, but they must be connected to the broader architecture that produces stable psychometric *g* and preserves relative structural profiles across domains.

## Figures and Tables

**Table 1:** Descriptive value ranges used to summarize each form of ergodicity.

Type of Ergodicity	Very High	High	Moderate	Weak	None
Variability ratio	0.80–1.25	0.60–0.79 or 1.26–1.67	0.40–0.59 or 1.68–2.50	0.20–0.39 or 2.51–5.00	<0.20 or >5.00
Structural r	≥0.90	0.75–0.89	0.50–0.74	0.30–0.49	<0.30 or negative
Structural MAD	≤0.10	0.11–0.20	0.21–0.35	0.36–0.50	>0.50
Dynamic r	≥0.90	0.75–0.89	0.50–0.74	0.30–0.49	<0.30 or negative
Averaging discrepancy	≤0.10	0.11–0.25	0.26–0.50	0.51–0.75	>0.75

Note: These ranges are descriptive interpretive guidelines rather than formal decision boundaries. For variance balance, parity is 1. For profile correlations, higher values indicate closer relative ordering; for MAD and averaging discrepancy, lower values indicate closer correspondence. Conclusions are based primarily on continuous estimates, bootstrap intervals, whole-matrix permutation tests, and matched finite-sample nulls.

**Table 2:** Peak starting age and approximate high-gain window for each process.

Process Family	Peak Starting Age	Approximate High-Gain Window
Working memory	≤4.3	4.3–5.3
Perceptual/inferential cognizance	≤4.3	4.3–4.5
Awareness of cognitive processes	≤4.3	4.3–7.4
Attention	6.6	5.7–8.0
Raven reasoning	6.9	5.2–9.1
Spatial reasoning	6.9	5.7–8.9
Deductive reasoning	8.2	7.0–9.4
Mathematical reasoning	≥17.2	16.2–17.2

**Table 3:** Main findings across the four studies.

Cross-Study Question	Study 1	Study 2	Study 3	Study 4
Does rank stability persist over time?	Raw stability reflects age; residual stability is process-specific	High locally in the model panel; diminishes with lag	Substantial over adjacent years; lower across two years; stronger in the second interval	Moderate locally; declines strongly with session lag
Does cross-sectional structure reproduce within-person structure?	Partial after age adjustment; magnitude weak	Strong adjusted profile in selected panel; observed two-wave result is cross-level change correspondence	Moderate task profile correspondence; pooled within-person magnitude very weak	Attention ordering partly preserved; broader and person-specific dynamics differ
Do trajectories self-average?	Partial over one year; attention differentiates; conditional panel near matched AR(1) expectation after detrending	Observed short-term convergence; generated raw dispersion increases but adjusted matched-null ratio is near parity	Three-wave dispersion close to matched AR(1) expectation	Exceeds homogeneous AR(1) null; random-intercept null accounts for terminal excess
Where do transition windows occur?	Age-specific maxima in one-year gain; attention window most robust	Threshold-dependent model-implied local-transition candidates in a minority	Not localizable with three annual waves; cross-interval replication tested instead	Observed training-related transition points are heterogeneous and subset-specific
Are individual differences in correspondence stable or predictive?	Continuous conditional distribution; negligible first-half/second-half stability	Not estimated independently of the generated panel	Moderate within-interval alignment, but no cross-interval stability or later-gain prediction	High local loading congruence, but no early–late stability or prediction of later learning rate
Main contribution	Age structure, developmental change, and conditional person-level correspondence	Conditional phase and coupling consequences of a generator	Observed cross-interval validation and real Wave-3 prediction	Microgenetic organization, idiographic heterogeneity, and temporal granularity

## Data Availability

De-identified data, analysis scripts, machine-readable outputs, and model provenance files are available from the corresponding author and will be deposited with the accepted article, where permitted.

## Supplementary Materials

The following supporting information can be downloaded at: https://www.mdpi.com/article/10.3390/jintelligence14100235/s1, Supplementary S1–S8 provide formal definitions, model-generation details, resampling and multiplicity procedures, reliability and prediction results, model-panel sensitivity analyses, idiographic benchmarks, transition-point robustness tests, the three-wave Study 3 validation, and person-level correspondence analyses across Studies 1, 3, and 4.
