# Supplementary material for: Beyond Structure and Change: Ergodicity and the Dynamic Organization of Intellectual Development
Source: J Intell. 2026 Oct 1;14(10):235. doi: 10.3390/jintelligence14100235 (PMC13706124; doi:10.3390/jintelligence14100235)
Supplement: Supplementary file 1 [file jintelligence-14-00235-s001.zip › jintelligence-4514356-supplementary.pdf]

Supplementary Materials

Beyond Structure and Change: Ergodicity and the Dynamic Organization of Intellectual Development

S1. The Four Forms of Ergodicity Tested in the Studies

The concept of ergodicity originated in statistical mechanics and probability theory, where it referred to equivalence between ensemble averages and time averages. A system is strictly ergodic when observations across many units at a point in time provide the same statistical information as observations of a unit across time. In psychology, Molenaar and colleagues emphasized that this equivalence requires stationarity and homogeneity, conditions rarely satisfied in development.

The four-form organization distinguishes variability, structural, dynamic, and averaging correspondence. The empirical indices diagnose different implications of cross-level equivalence; they are not interchangeable labels for strict ergodicity and are not presented as four new ergodic theorems. Their contribution lies in their joint application across developmental systems, individuals, ages, and temporal scales.

Variability Ergodicity

Variability ergodicity concerns the relation between variability observed within individuals over time and variability observed between individuals at a developmental period. The theoretical time/ensemble notation and empirical within/between estimators are bridged explicitly:

$$\sigma^2T = E_i[Var_t(X_it)] \;\equiv\; \sigma^2W$$

$$\sigma^2E = E_t[Var_i(X_it)] \;\equiv\; \sigma^2B$$

$$E_v = \sigma^2W \,/\, \sigma^2B$$

Complete dispersion parity is E_v=1. Values below 1 indicate greater between-person dispersion; values above 1 indicate greater within-person dispersion. Rank persistence and ICC-like stable variance are reported separately because they are not algebraically identical to the dispersion ratio.

$$r_{rank}(t,h) = cor_i(X_it,\, X_i,t+h)$$

Structural Ergodicity

Structural ergodicity concerns correspondence between relations among processes across individuals and relations among processes within individuals across repeated observations. For intensive data, the relevant matrices are:

$$\Sigma W = [Cov_t(X_j,\, X_k)]$$

$$\Sigma B = [Cov_i(X_j,\, X_k)]$$

Equation 8 defines the between-person structure, ΣB.

$$D_s = ||\Sigma W - \Sigma B||F$$

The empirical paper primarily compares correlation matrices. The profile coefficient and mean absolute discrepancy are:

$$rprofile = cor[vech(RB),\, vech(Rtarget)]$$

$$MAD = (1/m)\, \Sigma_e\, |rB,e - rtarget,e|$$

The profile coefficient measures relative ordering, whereas MAD measures absolute discrepancy. Whole-matrix bootstrap and joint row/column permutation are used because matrix edges are dependent.

Dynamic Ergodicity

Dynamic ergodicity concerns correspondence between cross-sectional organization and the organization of developmental change. In a dense series, increments may be defined within persons over many occasions. In Studies 1 and 2, the empirical target is instead the covariance or correlation across children in their W1-W2 changes:

$$\Delta X_{ij} = X_{ij},2 - X_{ij},1, \qquad R\Delta = cor_i(\Delta X_i1, \ldots, \Delta X_ip)$$

These two-wave matrices describe between-child covariance of developmental change and are not person-specific temporal covariance processes. They are therefore referred to as developmental-change correspondence.

Averaging or Self-Averaging Ergodicity

Averaging ergodicity asks whether cumulative individual histories become increasingly similar to the population trajectory. For standardized or detrended score Z:

$$\bar{Z}_i(t) = (1/t)\ \Sigma_{s=1}{}^{t}\ Z_i s$$

$$\Omega(t) = (1/N)\ \Sigma_i\ [\bar{Z}_i(t) - \bar{Z}\cdot(t)]^2$$

Because finite autocorrelated trajectories do not yield zero dispersion, the matched-null ratio is:

$$Q\Omega(t) = \Omega obs(t)\ /\ E[\Omega null(t)]$$

QΩ=1 indicates agreement with the specified finite-sample null. Interpretation depends on whether the null preserves only homogeneous serial dependence or also stable child-specific levels.

S2. Generation, Validation, and Interpretive Status of the Model-Based Ten-Wave Study 1 Dataset

Purpose and empirical basis

Study 1 included 425 participants aged 4.33-17.17 years at Wave 1, assessed twice at an interval of approximately one year. The combined age support of the observed waves extended to 18.01 years. The extension used 26 task scores grouped into eight cognitive families. Three infeasible Wave-2 awareness-of-cognitive-processes values were treated as missing in estimation; no observed Wave-1 or Wave-2 score was replaced.

The extension proceeded in two stages. First, the 425 original participants were extended recursively to ten waves. Second, 875 synthetic cases balanced the 13 integer starting-age cohorts from 4 through 16 years at 100 participants each, yielding model-based N=1,300. Provenance fields distinguish observed values, generated waves, and fully synthetic records.

Table S1. Cognitive families, indicators, longitudinal-gain weights, and phase-shift dispersion.

Cognitive family	Task indicators	λf	Phase-shift SD (years)
Attention	Compatible and incompatible Stroop-like tasks	.00	.24
Working memory	Word and pseudoword recall	.20	.83
Perceptual/inferential cognizance	Visual and auditory perceptual awareness; inferential awareness	.30	.82
Awareness of cognitive processes	Representational-, rule-, and principle-based awareness	.40	.94
Deductive reasoning	MP, MT, AC, DA	.50	.34
Mathematical reasoning	Four levels	.60	.83
Spatial reasoning	Four levels	.80	1.06
Raven reasoning	One- to four-dimensional matrices	1.00	.83

Stage 1: Temporal extension

For each task p, a nonlinear age-performance function and a one-year gain function conditional on starting score, age, and gender were estimated from the two observed waves. The recursive gain blended the age-curve increment with the directly observed gain:

$$\tilde{G}_p(a) = (1-\lambda f)[\mu_p(a+\Delta a)-\mu_p(a)] + \lambda f\ Gobs_p(a)$$

For participant i, task p, family f, and wave t, the latent next score was generated as:

$$Y^*{}_i p,t+1 = Y_i p,t + \tilde{G}_p(a^*{}_i f,t) + \beta_p[Y_i p,t-\mu_p(a^*{}_i f,t)] + \gamma_p Gender_i + \varepsilon_i p,t$$

Family-specific phase shifts were inferred from each participant's Wave-1 age-residual profile and bounded to exclude implausible offsets. Innovations were sampled jointly to preserve observed cross-task covariance of one-year change. Task-specific transformations enforced feasible ranges, floors and ceilings, and score support.

Three models were examined: a homogeneous null, an empirical-transition model, and the primary phase-shift model. Two hundred ten-wave panels were generated under each model. Waves 1-2 remained observed. Replication 120 of the phase-shift model was selected for illustration because it was closest to the ensemble median on the prespecified diagnostics.

Validation and age support

Five-fold cross-validation predicted held-out Wave-2 scores from Wave 1, age, and gender. Nested cross-validation confirmed moderate-to-strong one-year prediction but did not establish external validation of Waves 3-

10. Ages beyond 18.01 years were treated as extrapolation; gains were bounded and attenuated toward zero. A conservative file retained original participants remaining inside observed age support through Wave 10.

Stage 2: Cohort balancing

Each synthetic case used two donor trajectories from the same starting-age cohort and gender. A common interpolation weight λs~Beta(2,2), truncated to .15-.85, was applied to all tasks and waves:

$$YD_{s}p,t = \lambda sYd1,p,t + (1-\lambda s)Yd2,p,t$$

Weighted donor age was adjusted through task- and wave-specific age slopes. Residual variation added 5% of the relevant donor-pool task SD, and synthetic phase shifts used donor weights plus a perturbation of approximately 5% of the donor-pool phase-shift SD.

Sensitivity and interpretive status

The central analytic file permitted source-lineage, observed-age-support, provenance, and interpolation-weight sensitivity analyses. Fully synthetic trajectories were slightly smoother in raw adjacent-wave stability but slightly weaker in adjusted profile correspondence than original-participant projected trajectories. The full panel mean adjusted AR(1) ratio was 1.029, with a lineage interval [.998, 1.058]. These intervals are conditional on the selected panel and do not include uncertainty from refitting, model class, seed, or generator hyperparameters.

The original generator code and all 600 panels are required for complete sensitivity to λf, the Beta donor distribution, innovation noise, phase perturbation, model class, and computational runtime. The panel-generation runtime was not preserved. Simulated waves and synthetic participants are not independent empirical observations and do not contribute inferential precision as if N=1,300 were observed.

S3. Common Resampling, Multiplicity, Measurement, and Predictive Procedures

Complete participants were the resampling unit. Studies 1 and 2 retained both observed waves for every selected child. Study 3 retained all three observed annual waves for every selected child. Study 4 retained each child's complete 30-session series. Bootstrap distributions were used for rank stability, dispersion ratios, profile correlations, MAD, Ω ratios, peak ages, predictive contrasts, and cross-interval comparisons.

For matrix comparisons, the complete matrices were recalculated in every bootstrap sample. Exact family-label permutations were used when feasible; task matrices used 10,000 QAP-style joint row-and-column permutations. Benjamini-Hochberg FDR correction was applied within prespecified study-by-diagnostic families.

Observed two-wave age adjustment used cubic B-splines with primary df=5; gain-window sensitivity used df=3-7. Score-level measurement diagnostics included standardized alpha, change-score alpha, cross-wave correlation-structure similarity, and empirical floor/ceiling concentration. Item-level latent measurement invariance could not be tested.

Studies 1 and 2 used nested five-fold prediction of observed Wave 2. Study 3 estimated transition models from Waves 1-2 and evaluated them against the independently observed Wave 3. Study 4 fitted early-session models and predicted later real sessions.

S4. Study 1 Statistical Robustness and Prediction Analyses

Table S2. Observed Study 1 family diagnostics.

Family	Raw rank r	Adjusted rank r	95% CI adjusted	Within/between variance ratio	Ω(2)/Ω(1)
Attention	.303	.047	-.040 to .128	.537	1.452
Working memory	.609	.410	.329 to .488	.240	.744
Perceptual/inferential cognizance	.563	.314	.195 to .424	.281	.679
Awareness of cognitive processes	.705	.457	.370 to .535	.173	.720
Deductive reasoning	.858	.210	.104 to .314	.077	.907
Raven reasoning	.653	.305	.214 to .389	.211	.710
Mathematical reasoning	.700	.357	.256 to .452	.175	.900
Spatial reasoning	.661	.431	.349 to .508	.204	.819

Table S3. Observed Study 1 matrix correspondence.

Level	Comparison	Raw profile r	Adjusted profile r	Adjusted QAP q	Adjusted MAD
Family	Structural	.133	.504	.013	.102
Family	Wave 1 vs change	.374	.429	.029	.102
Task	Structural	.498	.809	<.001	.068
Task	Wave 1 vs change	.412	.741	<.001	.071

Table S4. Study 1 measurement and held-out prediction diagnostics.

Family	Change-score α	Held-out W2 r	Standardized RMSE	Interpretive qualification
Attention	.965	.357	.934	Reliable change but relatively weak prediction
Working memory	.093	.611	.791	Very weak change reliability
Perceptual/inferential cognizance	.267	.633	.773	Low change reliability
Awareness of cognitive processes	.458	.727	.686	Moderate change reliability
Deductive reasoning	.547	.924	.381	Highest prediction accuracy
Raven reasoning	.666	.737	.675	Moderate reliability
Mathematical reasoning	.377	.732	.681	Low-moderate change reliability
Spatial reasoning	.019	.699	.715	Change essentially unreliable

Study 1 outputs include full bootstrap distributions, empirical boundary diagnostics, spline-degree sensitivity, nested-CV hyperparameters and contrasts, lineage intervals, provenance contrasts, and period-by-family matched-AR(1) ratios.

S5. Study 2 Statistical Robustness and Model-Panel Analyses

Table S5. Observed Study 2 family diagnostics.

Family	Raw rank r	Adjusted rank r	95% CI adjusted	Within/between variance ratio	Ω(2)/Ω(1)
Attention	.570	.139	.011 to .270	.275	.648
Working memory	.636	.256	.138 to .372	.228	.832
Relational integration	.561	.181	.023 to .332	.281	.765
Raven reasoning	.393	.013	-.120 to .142	.442	.939
Cognizance	.227	.019	-.085 to .116	.621	.651

Table S6. Observed Study 2 matrix correspondence.

Level	Comparison	Profile r	95% bootstrap CI	QAP q	MAD
Family	Observed level-versus-change	.736	.353-.895	Descriptive legacy coefficient	.322
Family	Adjusted structural	.927	Bootstrap in workbook	.011	.037
Family	Adjusted W1 vs change	.827	Bootstrap in workbook	.011	.056
Task	Adjusted structural	.921	Bootstrap in workbook	<.001	.045
Task	Adjusted W1 vs change	.829	Bootstrap in workbook	<.001	.062

Table S7. Study 2 measurement reliability and held-out Wave-2 prediction.

Family	α W1	α W2	Change α	Held-out W2 r	Standardized RMSE
Attention	.906	.910	.822	.663	.748
Working memory	.821	.654	.356	.677	.736
Relational integration	.839	.853	.660	.649	.760
Raven reasoning	.680	.691	.516	.506	.861
Cognizance	.835	.810	.775	.339	.941

Table S8. Study 2 conditional model-panel audit and interpretation.

Quantity	Estimate	Interpretation
Adjusted mean family rank, lag 1	.930	Very strong local continuity under the generator
Adjusted mean family rank, lag 9	-.117 [-.179, -.055]	Long-lag reversal
Adjusted structural profile r	.899	Relative structure preserved conditionally
Adjusted developmental-change profile r	.414	Much weaker than structural profile
Mean raw Ω(10)/Ω(1)	2.991 [2.630, 3.488]	Raw cumulative dispersion increases
Adjusted observed/AR(1) Ω ratio	.997 [.985, 1.009]	No general excess beyond matched serial dependence
Mean correlation of W9-W10 with observed W1-W2 increment	.963	Late increments strongly inherit the initial derivative
Late increment SD / initial increment SD	≈.12	Late change variance is strongly compressed
Wave-10 scores outside observed W1-W2 support	59.6% on average	Substantial score extrapolation
Wave-10 ages beyond observed support	100%	Complete chronological extrapolation

Table S9. Threshold sensitivity of Study 2 model-implied transition prevalence.

Threshold	Classified as clear
z ≥ 1.50	49.4%
z ≥ 1.645	40.2%
z ≥ 1.75	34.2%
z ≥ 1.96	24.6%
z ≥ 2.326	12.6%
z ≥ 3.00	2.9%
Max-search 95% threshold, z=3.297	1.0%
Max-search 99% threshold, z=3.999	.4%

Table S10. Primary fitted minima for the SARA-C process proxies.

Process proxy	Fitted minimum wave
Search	3.47
Align	3.39
Relate	1.70

Process proxy	Fitted minimum wave
Abstract	1.52
Cognize	1.73

The full machine-readable Study 2 outputs additionally document held-out indicator validation, age-support percentages, empirical score-range violations, derivative carry-forward, AICc growth-model comparisons, period-specific process coupling, provenance contrasts, and sensitivity to common versus group-specific adjustment.

S6. Study 3: Three-Wave Observed Validation

Study 3 included 313 children observed at three annual waves. Analyses were restricted to nine measures observed at every wave: perceptual focus, perceptual inhibition, conceptual control, verbal, digit, and spatial working memory, spatial and analogical inductive reasoning, and deductive reasoning. Simulated awareness scores were excluded.

Table S11. Study 3 observed domains and indicators.

Domain	Observed indicators
Attention	PF, PI, CCR
Working memory	V, D, S
Inductive reasoning	IND_S, IND_A
Deductive reasoning	DR

Table S12. Study 3 rank persistence and variance balance.

Domain	W1-W2 r	W2-W3 r	W1-W3 r	Lag-2 contrast, 95% CI	Within/between variance ratio
Attention	.759	.880	.691	-.129 [-.172, -.089]	.287 [.234, .366]
Working memory	.580	.829	.483	-.221 [-.280, -.163]	.584 [.467, .762]
Inductive reasoning	.695	.786	.570	-.169 [-.225, -.120]	.461 [.370, .584]
Deductive reasoning	.660	.785	.523	-.199 [-.251, -.146]	.523 [.426, .662]

Table S13. Study 3 pooled three-wave structural correspondence.

Level	Profile r	95% CI	Mean between r	Mean within r	MAD	QAP q
Domains	.774	[.086, .944]	.228	.029	.199	.1200
Tasks	.454	[.151, .610]	.127	.027	.131	.0032

Table S14. Continuity of residualized developmental change across the two real intervals.

Domain	Change 1-2 with change 2-3 r	95% CI	FDR q
Attention	-.057	[-.199, .085]	.950
Working memory	-.004	[-.115, .107]	.950
Inductive reasoning	-.038	[-.147, .079]	.950
Deductive reasoning	-.008	[-.127, .109]	.950

Table S15. Psychometric organization versus common developmental change.

Score system	PC1 variance	First eigenvalue	Parallel-analysis threshold	Retained
Psychometric W1	50.7%	2.028	1.199	Yes
Psychometric W2	41.0%	1.641	1.199	Yes
Psychometric W3	36.7%	1.468	1.199	Yes
Residualized change W1-W2	30.7%	1.229	1.199	Marginally
Residualized change W2-W3	29.4%	1.174	1.199	No

Table S16. Annual autoregression and prospective prediction of the real Wave 3.

Domain	AR W1-W2	AR W2-W3	Carry-forward RMSE	AR RMSE	Priority RMSE	Full RMSE
Attention	.788	.894	.421	.422	.422	.423
Working memory	.571	.838	.569	.615	.617	.621
Inductive reasoning	.688	.855	.570	.583	.585	.585
Deductive	.638	.790	.626	.614	.619	.619

Domain	AR W1-W2	AR W2-W3	Carry-forward RMSE	AR RMSE	Priority RMSE	Full RMSE
reasoning						

No off-diagonal cross-domain annual transition path survived FDR correction. Ordered-priority, reverse-order, and unrestricted cross-domain models did not improve prediction of the real third wave over carry-forward or target-domain autoregression.

Table S17. Three-wave cumulative dispersion relative to the matched homogeneous AR(1) expectation.

Domain	Ω(2)	Ω(3)	95% CI for Ω(3)	AR(1) expected	Observed/null ratio
Attention	.879	.851	[.713, .989]	.846	1.005
Working memory	.790	.754	[.631, .874]	.756	.996
Inductive reasoning	.848	.789	[.666, .922]	.784	1.006
Deductive reasoning	.830	.771	[.655, .901]	.770	1.001

Comparison with Study 4. Study 3 and the three-anchor portion of Study 4 both produced very weak average within-person coupling across broad cognitive systems (approximately .03), whereas the 30-session attention core showed substantially stronger coupling within a narrow trained system.

S7. Study 4 Intensive and Idiographic Analyses

Table S18. Rank stability and dispersion diagnostics for the five dense Study 4 series.

Construct	Lag 1	Lag 15	Lag 29	Lag-29 q	ICC	Within/between SD ratio
PIC	.661	.452	.333	.003	.512	.694
CIC	.708	.438	.316	.003	.516	.802
ONM	.654	.486	.259	.003	.534	.822
PIC self-evaluation	.518	.244	.069	.448	.300	.819
ONM self-evaluation	.526	.251	.079	.448	.306	.805

Table S19. Structural correspondence under increasingly strict within-person decompositions.

Set	Estimator	Mean between r	Mean within r	MAD	Profile r	Matrix q
Attention core	Person-centered	.721	.457	.264	.991	.286
Attention core	Two-way centered	.721	.321	.399	.978	.286
Attention core	Individual trend + occasion	.721	.275	.445	.979	.286
Attention core	AR(1) innovations	.721	.245	.476	.974	.286
Core + self-evaluation	Person-centered	.524	.253	.271	.901	.050
Core + self-evaluation	AR(1) innovations	.524	.103	.421	.884	.050
Attention increments	Net gains vs adjacent increments	.355	.183	.172	.997	.286
Five-variable increments	Net gains vs adjacent increments	.208	.074	.134	.818	.050

Table S20. Time-average dispersion under homogeneous and random-intercept AR(1) nulls.

Construct	Observed Ω(30)	95% CI	Homogeneous AR(1) ratio	q	Random-intercept AR(1) ratio	q
PIC	.528	.461-.585	3.522	<.001	.991	.644
CIC	.533	.488-.575	3.028	<.001	.984	.644
ONM	.542	.496-.584	3.691	<.001	.982	.644
PIC self-evaluation	.326	.249-.400	3.264	<.001	.982	.644
ONM self-evaluation	.329	.270-.392	3.235	<.001	.978	.644

Table S21. Three-anchor domain measurement and correspondence diagnostics.

Domain	ICC	Mean between r	Mean within r	MAD	Profile r	α S1/S15/S30
Attention anchors	.361	.612	.336	.276	.999	.596/.769/.833
Working memory	.403	.256	.048	.207	1.000	.356/.432/.354
Relational complexity	.366	.725	.534	.191	.969	.887/.875/.873
Cognizance	.447	.691	.428	.264	1.000	.756/.818/.859
Language	.583	.446	.100	.345	-.678	.511/.693/.637
Raven levels	.194	.218	.066	.151	.855	.418/.438/.302
Mathematics	.482	.407	-.206	.613	—	-.021/.581/.313

Table S22. Prospective prediction of later observed sessions.

Construct	Carry-forward	Linear recursive	Quadratic recursive	Quadratic one-step
PIC	1.054	.898	1.228	.835
CIC	1.070	.912	1.030	.741
ONM	.977	.960	1.415	.914
PIC self-evaluation	1.115	1.060	1.140	.935
ONM self-evaluation	1.191	1.136	1.110	.921

Table S23. P-technique and VAR benchmarks.

Method	Set	Central estimate	Heterogeneity / reliability	Implication
P-technique covariance	Attention core	Median profile=.929	IQR .714-.973; odd-even=.831	Common ordering, not universality
P-technique covariance	All five	Median=.663	IQR .442-.784; odd-even=.587	Self-evaluation increases heterogeneity
P-technique factor	Attention core	Median Tucker=.973	90.3% >.85; odd-even=.940	Strong common attention loading pattern
P-technique factor	All five	Median=.798	40.0% >.85; odd-even=.722	Substantial person-specific variation
Pooled VAR innovations	Between vs temporal	Profile=.880; means .393 vs .104	QAP p=.0165	Relative order retained; magnitude weaker
Person-specific ridge VAR	Person vs pooled	Median=.277; IQR .164-.394	Only 5.2% >.50	Pooled dynamics poorly represent most children

Table S24. Selection-aware training-transition prevalence.

Construct	Mean candidate session	SD	Above 95% null	q	Above 99% null	q
PIC	15.70	5.87	13.5%	.0013	3.2%	.031
CIC	17.08	5.05	18.1%	.0013	3.2%	.031
ONM	15.79	5.92	9.0%	.026	1.9%	.205
PIC self-evaluation	12.85	6.25	20.6%	.0013	5.2%	.005
ONM self-evaluation	13.55	6.05	20.0%	.0013	3.2%	.031

Table S25. Event-aligned peaks relative to selected-null peaks.

Construct	Observed peak	Selected-null peak	q	Conclusion
PIC	1.375	1.239	.0075	Exceeds selection-only expectation
CIC	1.303	1.276	.287	No evidence beyond selection
ONM	1.278	1.209	.057	Marginal
PIC self-evaluation	1.494	1.250	.005	Exceeds selection-only expectation
ONM self-evaluation	1.358	1.251	.0083	Exceeds selection-only expectation

Across 64 robust models, no transition timing or magnitude predictor survived FDR correction for transfer to untrained cognitive domains.

S8. Individual Differences in Cross-Level Correspondence

The person-level analyses use design-specific correspondence measures. These measures quantify how closely an individual trajectory or change vector resembles a population reference over a specified interval. They are not trait estimates of strict ergodicity. Study 1 is conditional on the selected ten-wave generated panel; Studies 3 and 4 use only observed data.

Study 1: conditional ten-wave individual correspondence

For each starting-age cohort, a leave-one-out Wave-1 correlation profile among the eight cognitive families defined the cross-sectional reference. For participant i, rS_i was the correlation between this reference and the individual ten-wave family-pair correlation profile; $r\Delta_i$ was the corresponding correlation for the nine adjacent-increment profile. The combined score was $I_i = 50\{1 + \tanh[(z(rS_i)+z(r\Delta_i))/2]\}$. Age-, sex-, and provenance-adjusted family scores were used. The bottom and top quintiles were used only for descriptive figures.

Table S26. Study 1 individual cross-level correspondence.

Statistic	Estimate	95% interval
Mean score, full conditional panel	52.17	51.41-52.98
SD and range	9.37; 26.73-84.06	-
Structural-increment component correlation	.694	-
Original-participant projected mean	51.90	50.98-52.85
Fully synthetic mean	52.30	51.52-53.14
Synthetic minus original	.40	-.27 to 1.03
First-half versus second-half score correlation	.016	-.058 to .093
Correlation with starting age	-.035	-.120 to .044
20th and 80th percentile cut points	43.89; 59.93	-

Table S27. Family-specific alignment by Study 1 correspondence group.

Family	Bottom 20%	Middle 60%	Top 20%
Attention	-.137	.058	.252
Working memory	-.209	.002	.217
Perceptual/inferential cognizance	-.220	-.007	.291

Family	Bottom 20%	Middle 60%	Top 20%
Awareness of cognitive processes	-.176	.036	.255
Deductive reasoning	-.205	.037	.221
Raven reasoning	-.179	.063	.304
Mathematical reasoning	-.240	.015	.318
Spatial reasoning	-.206	.061	.332

The score distribution was approximately symmetric and unimodal (descriptive omnibus normality p=.222). It was not related to starting age and did not differ reliably between original-participant projected and fully synthetic trajectories. Its negligible first-half/second-half stability indicates that it is a time-local model-based index rather than a stable personal trait.

Study 3: individual alignment across real annual intervals

Each child's baseline-standardized nine-task change vector was compared, by cosine similarity, with the leave-one-out mean change vector of the child's starting-age cohort. Alignment was calculated independently for Wave 1-Wave 2 and Wave 2-Wave 3. Prediction of later mean gain controlled prior mean gain, baseline general level, age, and sex using HC3 robust inference.

Table S28. Study 3 individual developmental alignment.

Statistic	Estimate	95% CI	p
Mean alignment, W1-W2	.477	.447-.505	-
Mean alignment, W2-W3	.505	.480-.529	-
Cross-interval stability	-.042	-.161 to .089	.463
Early alignment predicting later mean gain, standardized β	-.046	-.224 to .132	.614

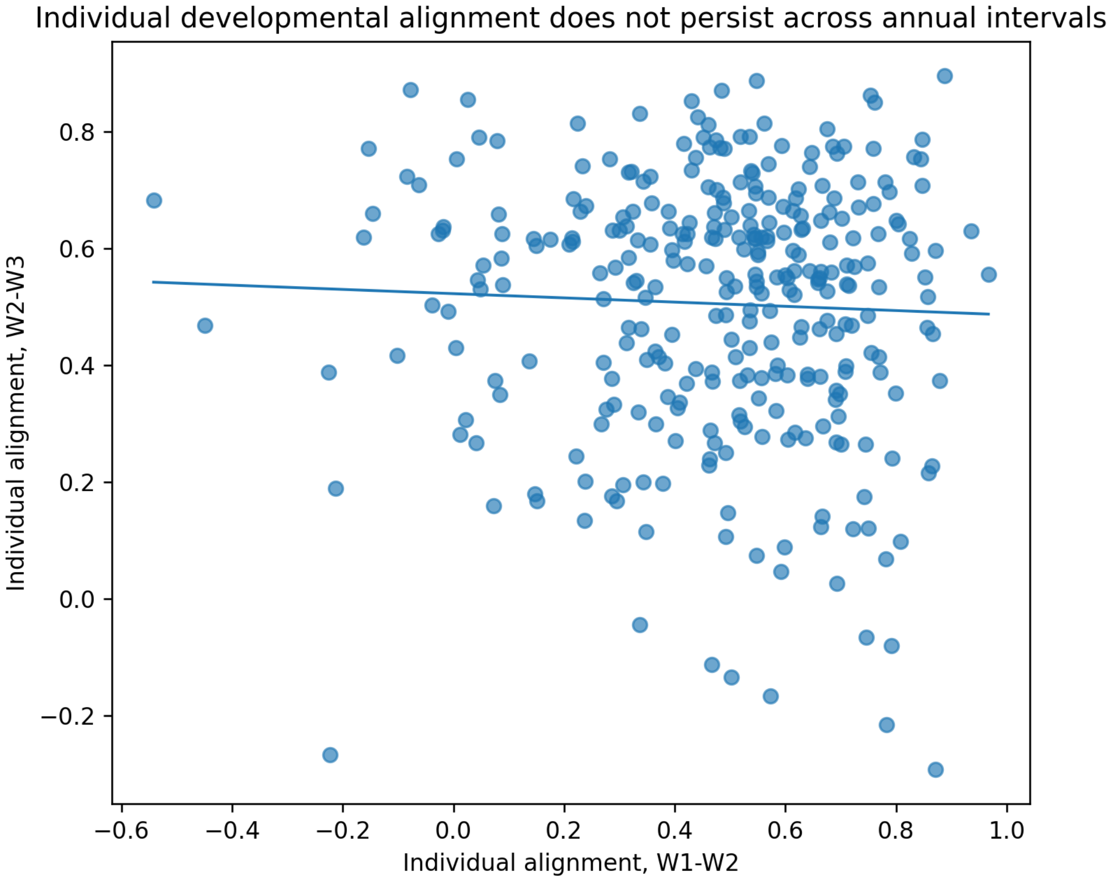

Figure S1. Individual alignment with the age-cohort change vector does not persist from the first to the second annual interval in Study 3.

Study 4: individual attention congruence and later learning

The three observed attention-performance series were standardized and residualized for common occasion effects and child-specific linear trends. Each child's first principal-component loading pattern during Sessions 1-15 was compared by Tucker congruence with the age- and gender-adjusted between-person loading pattern. Congruence was repeated for Sessions 16-30. The later composite learning slope was regressed on early congruence while controlling early slope, Session-15 performance, age, and gender with HC3 robust inference.

Table S29. Study 4 individual loading congruence and later learning.

Statistic	Estimate	95% CI	p
Median congruence, Sessions 1-15	.950	.917-.969	-
Median congruence, Sessions 16-30	.983	.969-.989	-
Early-late stability of congruence	-.072	-.207 to .093	.372
Early congruence predicting later learning slope, standardized β	-.086	-.218 to .047	.204

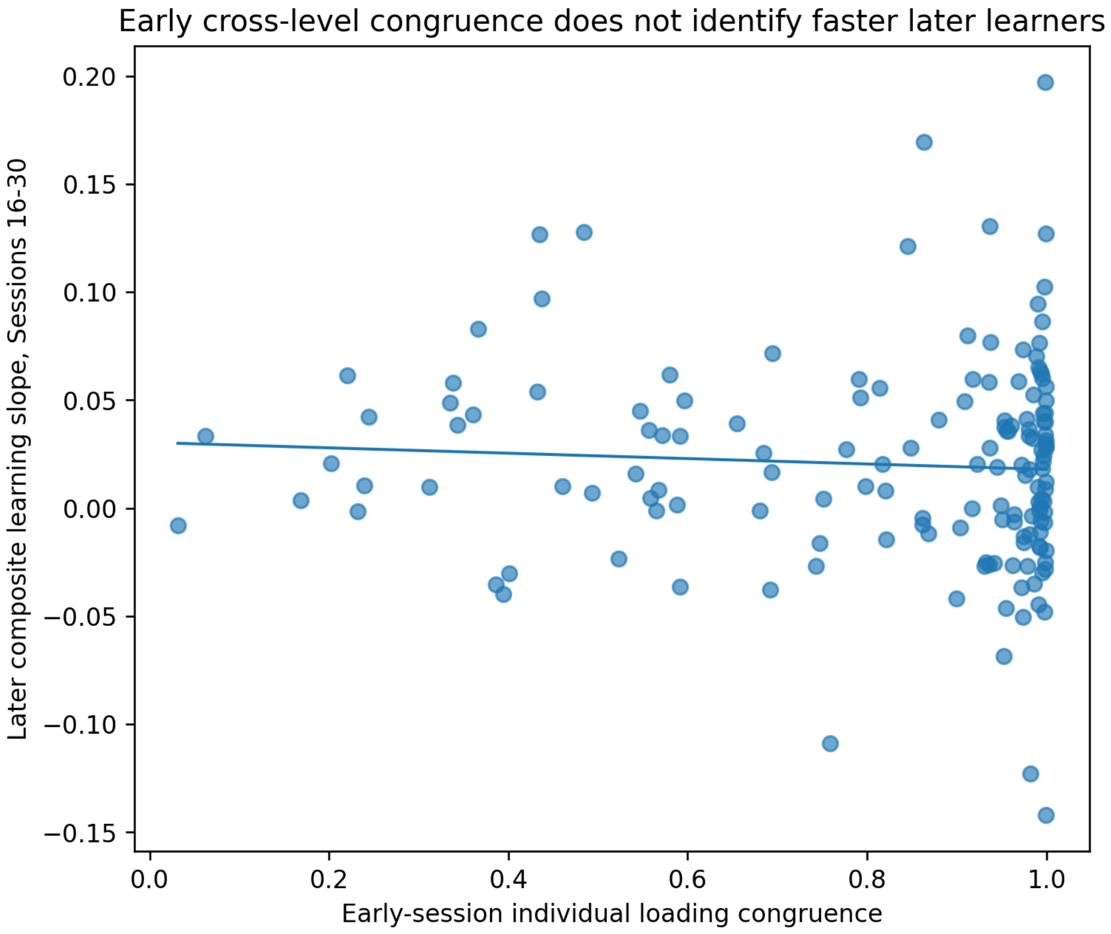

Figure S2. Early-session loading congruence does not identify faster subsequent learners in Study 4.

Across Studies 1, 3, and 4, person-level correspondence was meaningful within a specified interval but showed little temporal rank stability and no prospective relation to later developmental or learning rate. These findings support a state-like, phase-dependent interpretation rather than a fixed individual ergodicity trait.

S9. SARA-C, Psychometric g, Developmental Generality, and Developmental g: Supporting Analyses

SARA-C as an Overlapping Developmental-Priority System

The empirical tests linked to Study 2, Study 4, and Section 3.5 treat SARA-C as an integrated and cumulatively expanding meaning-making mechanism rather than as a rigid serial chain. Search, Align, Relate, Abstract, and Cognize are functionally distinguishable operations of one system. All operations available at a given developmental level may contribute to performance; what changes with age and phase is their relative developmental priority—the operation or coupling contributing most strongly to acceleration, coordination, propagation, or predictive reach. The model therefore predicts overlapping developmental windows and changing process centrality, not an annual-wave sequence in which one SARA-C operation must be completed before the next becomes active.

Search–Align was indexed by attention focus, selective attention, and inhibition; Relate by relational-integration tasks of increasing complexity; Abstract by complex Raven, inductive, deductive, mathematical, or spatial reasoning; and Cognize by perceptual awareness (PA), inferential awareness (IA), awareness of cognitive processes (ACP), and, more provisionally, self-evaluation. Working memory was treated as an enabling resource rather than relabeled as a SARA-C operation. Priority was evaluated through converging evidence from age-specific gain functions, loadings on interval common-change dimensions, coordination or centrality of changes, transition timing, and held-out prediction. Original participants and observed waves or sessions supplied the empirical evidence; findings from generated trajectories were interpreted only as conditional consequences of the fitted models.

Table S30. Cross-study evaluation of the overlapping-priority SARA-C hypotheses.

Hypothesis	Most supportive evidence	Limiting evidence	Evaluation
Available operations remain integrated in attained organization	Broad psychometric g was retained at all observed waves; most state loadings were positive.	Some windows were multidimensional and measurement quality changed.	Supported at the level of attained organization.
Search–Align has early developmental priority	Study 1 Search and Align peaked near 6.5 and 6.8 years and dominated early common change; Study 4 learning was fastest early.	Study 2 peaks were lower-bound estimates; not every attention indicator had identical timing.	Supported with qualification.
Relate becomes a central carrier in middle childhood	In Study 2, Relate had the strongest developmental loading at ages 5–8 (.758) and remained high at 7–10 (.633).	Mean Relate gain peaked at the lower boundary and attention remained active.	Partially supported; central carrier rather than exclusive stage.
Abstract expands with complexity and age	Study 1 Raven and mathematics levels tended to peak later; Study 4 higher relational-complexity levels became active later.	The ordering was not universal in spatial, Study 2 Raven, or the highest Study 4 Raven level.	Partially supported; complexity-dependent and domain-specific.
Cognize becomes explicit through PA, IA, and ACP	Study 2 PA tended to precede IA; Study 4 PA related more strongly than IA to attention at Session 1.	Study 1 awareness peaks were boundary-dominated; Study 3 awareness was simulated; Study 4 lacked dense PA/IA/ACP.	Weak or insufficient.
Developmental windows overlap	Study 1 high-gain regions overlapped widely; Study 4 attention transition timing was closer than a permutation null.	Several estimates were boundary-dominated.	Supported as probabilistic overlapping windows.
Construction is followed by canalization and differentiation	Study 4 slopes declined from .084 to −.006 and adjacent rank persistence increased from .646 to .725.	Cumulative trajectory dispersion also increased from .592 to .680.	Partially supported; stabilization of ordering can coexist with trajectory differentiation.
Developmental generality changes composition by phase	Different processes carried gΔ across studies and Study 4 intervals; Study 3 developmental scores did not persist.	Batteries and reliabilities differed across studies.	Broadly supported.
Cognize improves correction or transfer	PA showed selective concurrent relation to attention.	Prospective self-evaluation feedback and transfer effects were nonsignificant after correction.	Not supported by the present proxies.

Table S31. Held-out prediction of observed change under alternative developmental-priority models.

Study	Invariant	Overlapping priority	Strict stage	Unconstrained smooth	Best model
Study 1	.8015	.7995	.8008	.7941	Unconstrained smooth
Study 2	.7720	.7694	.7672	.7721	Strict stage (small advantage)
Study 3	.5900	.5901	.5908	.5914	Invariant (small advantage)

Values in Table S31 are mean standardized RMSE; lower values indicate better prediction. Differences were small and the prespecified overlapping-priority model was not uniformly superior. The results therefore support SARA-C as a cumulative and probabilistic framework that organizes testable age- and phase-sensitive hypotheses, but they do not uniquely identify it as the causal architecture underlying the observed transitions.

Constructs, Score Construction, and Factor Extraction

Section 3.5 distinguishes three levels of generality. Psychometric g, gP(t), is the main common dimension of attained cognitive levels at a specified occasion. Interval developmental generality, gΔ(t1,t2), is the common covariance among changes after prior level, age, and sex or gender are taken into account. Developmental g in the stronger mechanistic sense, gD,i(t), refers to the person- and phase-dependent efficiency with which cognitive organization is updated, coordinated, consolidated, propagated, and transferred. A factor extracted from one interval of change is therefore treated as an empirical projection of a possible developmental operator, not automatically as the operator itself.

The cross-study analyses prioritize original participants and observed waves or sessions. Studies 1 and 2 contribute one observed interval of between-child covariance in residualized change; Study 3 contributes two successive real annual intervals and a supplementary three-wave developmental direction; Study 4 contributes three broad cognitive assessments and dense 30-session attention and self-evaluation series. Simulated awareness scores in Study 3 were excluded, and generated waves and synthetic participants were not used as independent evidence for the psychometric-developmental conclusions.

Domain scores and scaling. One composite per cognitive domain, rather than every individual task, entered the cross-study decompositions so that domains with more indicators did not receive disproportionate weight. Study 1 used eight domains (attention, working memory, perceptual/inferential cognizance, awareness of cognitive processes, deductive, Raven, mathematical, and spatial reasoning); Study 2 used five (attention, working memory, relational integration, Raven reasoning, and cognizance); Study 3 used four observed domains (attention, working memory, inductive reasoning, and deductive reasoning); and Study 4 used seven performance domains (attention, working memory, relational integration, cognizance, language, Raven reasoning, and mathematical reasoning). Scores were oriented so that higher values indicated better performance. Studies 1–3 used the archived domain composites defined from the same task sets at each observed wave. In Study 4, task indicators were standardized on a common pooled metric across Sessions 1, 15, and 30 and then averaged with equal weights within each domain; self-evaluation was examined separately in the primary model. Because all decompositions used correlation matrices, each domain entered with unit variance. Analyses used complete domain vectors for the variables entering a particular solution; the primary Study 4 anchor-session solutions used 154 children because one Wave-1 Raven score was missing.

Psychometric g. The primary operational estimate of psychometric g was the unrotated first principal component (PC1) of the Pearson correlation matrix of the standardized domain scores at each observed wave or anchor session. Thus, the term factor is used substantively in the manuscript, but the primary score extraction was principal component analysis rather than a confirmatory causal factor model. Let Zt denote the N × p matrix of standardized domain scores at occasion t and Rt its correlation matrix. The eigen-decomposition was:

$$R\_t = \mathrm{cor}(Z\_t) = V\_t\,\Lambda\_t\,V\_t'. \quad \text{(S5)}$$

The first eigenvector v1t was oriented so that the sum of its loadings was positive. Individual psychometric-g scores, component loadings, and variance summarized were calculated as:

$$g\_P{,}i(t) = z\_i(t)'v\_1t; \quad \ell\_pt = v\_p1{,}t\sqrt{\lambda\_1t}; \quad \%\mathrm{Var(PC1)} = 100\lambda\_1t/p. \quad \text{(S6)}$$

PC1 scores were used for cross-wave score stability and for the state-transition regressions. The PC1 solution was supplemented by an unrotated one-factor common-factor solution to evaluate common-factor reliability. In Study 4 this model was fitted by maximum likelihood; the corresponding archived one-factor solutions provided omega for Studies 1–3. Omega was computed from the one-factor loadings (ℓp) and uniquenesses (θp):

$$\omega = (\Sigma\_p\,\ell\_p)^2 \,/\, [(\Sigma\_p\,\ell\_p)^2 + \Sigma\_p\,\theta\_p]. \quad \text{(S7)}$$

Dimensional retention was evaluated by Horn parallel analysis. Observed correlation-matrix eigenvalues were compared with the 95th percentile of eigenvalues from random Gaussian datasets matched on sample size and number of domains. Study 4 used 5,000 Monte Carlo replications; the same 95th-percentile retention rule was used in the archived Study 1–3 analyses. Loading stability across occasions was quantified by Tucker congruence, and individual score stability by Pearson correlations between occasion-specific PC1 scores. Where reported, age-adjusted sensitivity solutions repeated the decomposition after removing age, age squared, and sex or gender from the domain scores.

Interval developmental generality. Developmental generality was not computed from raw difference scores alone. For each domain p and observed interval t1–t2, the later domain score was regressed on the prior score in the same domain, chronological age, age squared where available, and sex or gender:

$$X\_ip(t_2) = \alpha\_p + \beta\_pX\_ip(t_1) + \gamma\_1pAge\_i + \gamma\_2pAge\_i^2 + \delta\_pSex\_i + e\_ip(t_1{,}t_2). \quad \text{(S8)}$$

The residual eip(t1,t2) indexes whether a child advanced more or less than expected from the level already attained and from normative age-related change; it is therefore conditional relative advancement, not raw gain.

Residuals were standardized across children, assembled into an N × p residual-change matrix, and submitted to the same correlation-matrix PCA, one-factor reliability analysis, and Horn parallel analysis used for psychometric g. The first component score was denoted gΔ(t1,t2) only when the solution was sufficiently broad and retained; otherwise it was described as the leading developmental direction. Studies 1 and 2 each provided one W1–W2 solution. Study 3 provided separate W1–W2 and W2–W3 solutions and, as a supplementary summary, a first component of the children's domain-specific linear slopes across all three observed waves. Study 4 provided S1–S15, S15–S30, and S1–S30 solutions. When parallel analysis retained two dimensions, as in the second Study 4 interval, the PC1 was not interpreted as a unique scalar developmental g; a two-component varimax rotation was used only to describe the main routes of coordinated change.

Dense learning generality in Study 4. The intensive series was used to estimate local learning rather than broad interval change. For each child i and trained process q, an ordinary least-squares slope on session number was estimated separately for Sessions 1–15, 16–30, and 1–30:

$$Y\_iqs = a\_iq + b\_iq \cdot Session\_s + \varepsilon\_iqs. \quad (S9)$$

The correlation matrix of the three performance slopes (PIC, CIC, and ONM) was decomposed to estimate a local attention-learning component, and the two self-evaluation slopes were decomposed separately to index metacognitive change. The all-five-slope analysis tested whether performance learning and self-evaluation formed one dimension; parallel analysis instead retained two dimensions. Early and late learning-component scores were then correlated and tested prospectively against later learning, nontrained change, and later psychometric state.

Candidate developmental g in Study 4. A stronger developmental operator was tested rather than assumed. Four person-level components were constructed: (1) attention update, the early PIC–CIC–ONM learning-component score; (2) metacognitive change, the early self-evaluation slope-component score; (3) attention consolidation, the residual of Session-30 attention after Session-15 attention, early attention learning, age, age squared, and sex were controlled; and (4) nontrained transfer, the equal-weighted mean of standardized S15–S30 residual changes in working memory, relational integration, cognizance, language, Raven reasoning, and mathematical reasoning. These four components formed the candidate operator vector:

$$D\_i = [Update\_i, Monitoring\_i, Consolidation\_i, Transfer\_i]'. \quad (S10)$$

The correlation matrix of Di was subjected to the same PCA, parallel-analysis, and one-factor omega criteria. The first component explained 31.9%, but its eigenvalue (1.277) did not exceed the 95th-percentile parallel threshold (1.288), and omega was .293. Consequently, no scalar developmental-g factor was retained. In this paper, gD,i(t) therefore remains the stronger theoretical construct denoting the person- and phase-dependent operator of updating, monitoring, consolidation, propagation, and transfer; the Study 4 analysis was a direct test of one operational approximation, not an assertion that the operator had been successfully measured.

Retention, stability, and uncertainty. Percentage of variance summarized was never used alone to declare a general factor. Interpretation combined parallel-analysis retention, loading breadth and sign, one-factor omega, loading congruence, and the stability or prospective validity of individual component scores. Components were globally sign-oriented but mixed loadings were otherwise preserved. Complete children were resampled so that all observations belonging to a child remained together. Study 4 used 1,000 factor-extraction bootstraps and 3,000 path bootstraps; robust state-transition regressions used HC3 standard errors, and Session-30 prediction used 50 repetitions of 10-fold cross-validation. Because batteries and indicator aggregation differed across studies, percentages of variance are interpreted within each analysis and are not treated as strictly commensurate effect sizes.

Table S32. Distinguishing psychometric state, interval developmental generality, dense learning generality, and the developmental operator.

Construct	Symbol / estimate	Operational meaning	Evidence required for the stronger interpretation	Status in the present studies
Psychometric state	gP(t)	Common covariance among attained domain levels at one occasion.	Broad loadings, factor retention, reliable scores, and loading/rank stability across occasions.	Retained at every observed wave or anchor session.
Interval developmental generality	gΔ(t1,t2)	Common covariance among age- and prior-level-adjusted changes during one interval.	Broad reliable loadings, factor retention, and replication or lawful transformation across intervals.	Moderate in Study 2, weak in Study 1, marginal/absent across Study 3 intervals, and phase-specific in Study 4.
Dense attention-learning generality	Local learning factor	Common individual learning rate across PIC, CIC, and ONM within a training phase.	Coherent slopes and prospective prediction of later learning, consolidation, or transfer.	Coherent within each half of Study 4, but not positively stable across halves.
Developmental operator	gD,i(t)	Efficiency of update, monitoring, consolidation, propagation, and transfer.	A broad reliable factor that predicts later reorganization and transfer beyond current state.	Not retained as one scalar factor in Study 4; remains a theoretical construct.

Psychometric State and Developmental Generality across Studies 1–4

A broad psychometric factor was recovered at every observed testing occasion. Its loading configuration and individual score ordering were highly stable even where its share of total variance declined. In contrast, common change was consistently weaker and changed in dimensionality, loading composition, or individual membership across intervals. The results therefore support a state-transition distinction: psychometric g is a robust coordinate of organization already attained, whereas gΔ describes the temporary breadth and direction of coordinated change.

Table S33. Strength and stability of psychometric g in the observed portions of the four studies.

Study	Observed occasions	PC1 variance (%)	Omega	Loading congruence	Individual g-score stability
Study 1	W1, W2	58.4, 51.9	.898, .865	.970	r12=.891
Study 2	W1, W2	66.7, 59.3	.876, .830	.986	r12=.740
Study 3	W1, W2, W3	62.1, 52.7, 46.1	.798, .705, .612	>.98	r12=.878; r23=.908; r13=.809
Study 4	S1, S15, S30	35.3, 41.7, 38.8	.692, .766, .730	.981–.987	r1,15=.797; r15,30=.811; r1,30=.772

The psychometric-developmental synthesis uses the component solutions reported in Section 3.5 and in the corresponding analysis archive. Study-specific ergodicity tables elsewhere in this supplement may use different score constructions for a narrower purpose; estimates from different indicator sets should not be substituted for one another.

Table S34. Comparative evidence for interval developmental generality.

Study / interval	Variance summarized	Om ega	Factor status	Principal carriers	Relation to state or next interval
Study 1, W1–W2	22.4%	.484	Retained, weak	Mathematical, spatial, and Raven reasoning	Initial g with gΔ: r=.117
Study 2, W1–W2	41.0%	.637	Retained	Relational integration, working memory, Raven, attention	Initial g with gΔ: r=.045
Study 3, W1–W2	30.8%	.131	Marginal	Attention, deductive, and inductive reasoning	Interval scores did not persist
Study 3, W2–W3	28.7%	.049	Not retained	Primarily attention	gΔ12 with gΔ23: r=.009
Study 3, three-wave direction	29.9%	.181	Not retained	Attention-centered	Initial g with direction: r=.074
Study 4, S1–S15	25.5%	.496	Retained	Relational integration, language, Raven, attention	gP1→gΔ1: β=.360
Study 4, S15–S30	22.3%	.384	Two factors retained	WM–Relate–Raven and attention–language routes	gP15→gΔ2: β=.333; rΔ1,Δ2=.137
Study 4, S1–S30	23.9%	.446	Retained	Relational integration and Raven	Broad full-interval summary

The strongest cross-study contrast concerns prediction of change. Initial psychometric g was nearly unrelated to the principal developmental estimate in Studies 1–3 (r=.117, .045, and .074). Study 4 qualified this result: psychometric state predicted some breadth of cross-domain reorganization during structured learning, but it did not predict dense attention-learning or self-evaluation-learning rates. Thus, psychometric g predicts later standing strongly and may constrain broad reorganization under favorable conditions, but it is not equivalent to local learning speed or a stable developmental capacity.

Study 4: Prospective State-Transition Tests and the Developmental Operator

Study 4 permitted the most direct test of how interval developmental generality contributes to a later psychometric state. The primary performance model included attention, working memory, relational integration, cognizance, language, Raven reasoning, and mathematical reasoning. Self-evaluation was examined separately. The three anchor-session psychometric factors were broad and stable, whereas the developmental factors changed composition across the two training halves.

Table S35. Study 4 state-transition and dense-learning tests.

Outcome	Predictor / conditioning	Standardized estimate	95% CI or q	Interpretation
First-interval developmental generality	Session-1 psychometric g	β=.360	95% CI [.217, .503]	Psychometric state predicted some broad relative reorganization.
Second-interval developmental generality	Session-15 psychometric g	β=.333	95% CI [.146, .520]	The state-to-change association recurred, but was domain-sensitive.
Session-30 psychometric g	gΔ1 controlling Session-1 g	β=.263	95% CI [.159, .367]	Early broad change contributed positively to later standing relative to baseline.
Session-30 psychometric g	gΔ1 controlling Session-15 g	β=−.423	95% CI [−.563, −.283]	At the same intermediate state, greater earlier change indexed less remaining change.

Outcome	Predictor / conditioning	Standardized estimate	95% CI or q	Interpretation
Second-interval developmental generality	gΔ1 controlling Session-15 g	β=−.401	95% CI [−.656, −.147]	Early generalized change carried information about phase/deceleration.
Early attention learning	Session-1 psychometric g	β=−.032	q=.724	Psychometric g did not predict local early learning speed.
Late attention learning	Session-15 psychometric g	β=.056	q=.724	Psychometric g did not predict local later learning speed.
Early vs later attention-learning factors	Factor-score correlation	r=−.185	95% CI approximately [−.390, .034]	Learning generality was not positively stable across phases.
Cross-validated Session-30 g	Add gΔ1 to Session-1 g	ΔRMSE=−.074	Improved in 50/50 repetitions	Early gΔ added predictive information beyond baseline state.
Cross-validated Session-30 g	Add gΔ1 to Session-15 g	ΔRMSE=−.084	Improved in 50/50 repetitions	gΔ also added phase information beyond the intermediate state.

The positive baseline-referenced and negative current-state-conditioned coefficients answer different questions. The first asks whether early generalized change contributes to final standing beyond the starting state; the second asks which children continue changing after reaching the same intermediate state. Because gΔ1 and Session-15 g share information from Session 15, the negative coefficient may also contain part-whole or suppression effects. It is therefore interpreted as predictive information about developmental phase rather than as a causal inhibitory effect.

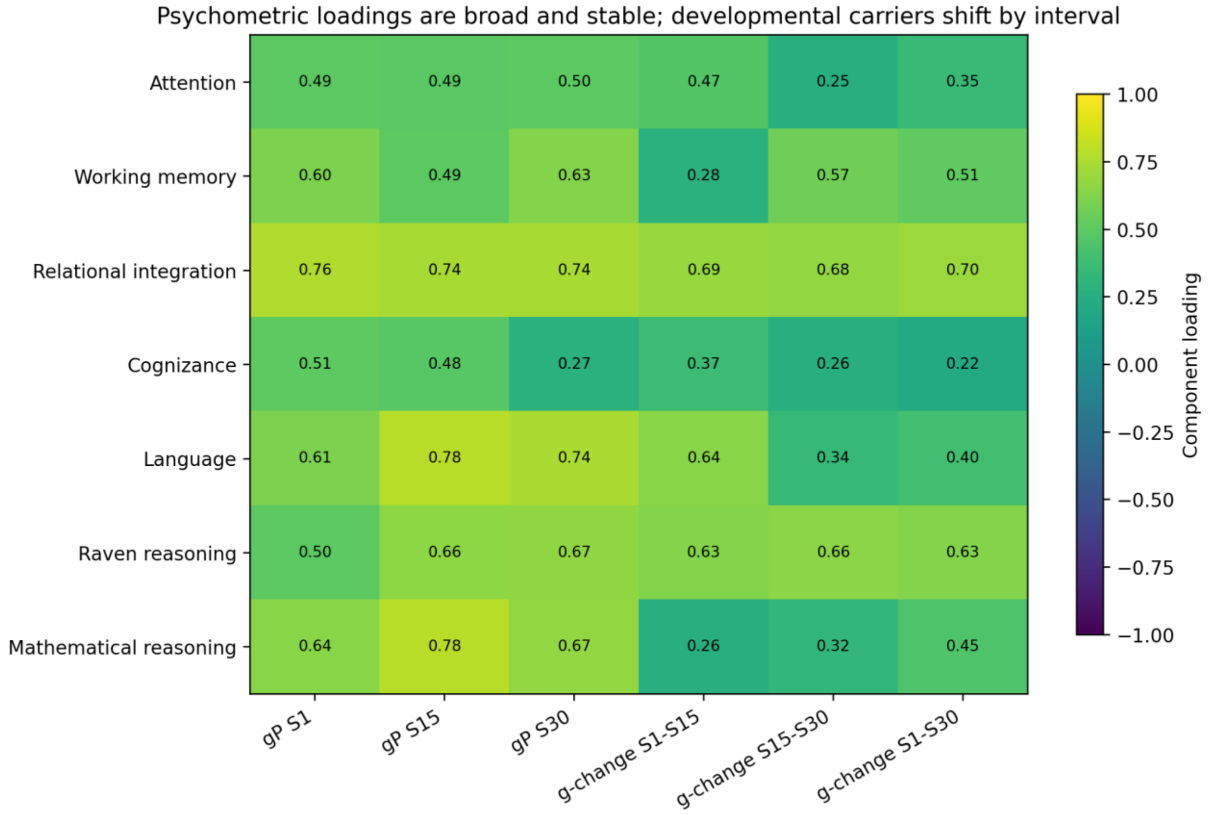

Figure S3. Domain loadings on psychometric g at Sessions 1, 15, and 30 and on the leading developmental dimensions for Sessions 1–15, 15–30, and 1–30 in Study 4. Psychometric loadings remain broad and relatively stable, whereas the processes carrying developmental generality shift by interval.

Table S36. Study 4 dense learning and candidate developmental-operator results.

Analysis	Variance / reliability	Retention or association	Conclusion
Early attention-learning factor, S1–S15	PC1=61.0%; ω=.722	One factor retained	PIC, CIC, and ONM formed a coherent local learning dimension.
Late attention-learning factor, S16–S30	PC1=66.9%; ω=.771	One factor retained	A coherent attention-learning dimension reappeared in the later phase.
All five dense slopes	PC1≈40%–42%	Two factors retained in both halves	Performance learning and self-evaluation change were not one dimension.
Candidate developmental operator	PC1=31.9%; eigenvalue=1.277; PA95=1.288; ω=.293	No factor retained	Update, self-evaluation, consolidation, and transfer did not form one scalar gD.
Partial SARA-C relay	Attention→Relate β=.181, p=.018, q=.184; later paths near zero	No relay path survived FDR	The available measures did not demonstrate an ordered attention–relation–reasoning–cognizance cascade.

Interpretive Synthesis

Across the four studies, psychometric g is a stable coordinate of attained cognitive organization, whereas developmental generality is weaker, interval-specific, and carried by different processes. Study 4 shows that interval developmental generality can improve prediction of a later psychometric state, but it does not behave as a stable growth trait and does not reduce to local attention-learning speed. The candidate developmental operator also failed to form a single reliable factor. These findings motivate a State–Transition–Priority account: the current psychometric state constrains the possibilities for change; developmental generality summarizes the temporary breadth of coordinated transition; and SARA-C, routed through phase- and person-dependent priorities, remains a candidate mechanism through which one state is transformed into the next.

The evidence is strongest for overlapping Search–Align development in early childhood, for a central role of relational integration in coordinated middle-childhood change, and for complexity-sensitive expansion of Relate and Abstract. Evidence for the developmental differentiation and feedback function of Cognize remains limited. Stronger identification of gD and SARA-C requires densely repeated, real measures of all operations; longitudinal measurement comparability; prospective prediction of independently observed transitions; and evidence that updating, monitoring, consolidation, propagation, and transfer cohere within persons or transform lawfully across phases.
